# High core 1β1,3-galactosyltransferase 1 expression is associated with poor prognosis and promotes cellular radioresistance in lung adenocarcinoma

**DOI:** 10.1007/s00432-024-05745-y

**Published:** 2024-04-25

**Authors:** Yong Chen, Yanyan Ji, Lin Shen, Ying Li, Yue Ren, Hongcan Shi, Yue Li, Yunjiang Wu

**Affiliations:** 1 Department of Medical Oncology, Affiliated Hospital of Yangzhou University, Yangzhou University, Yangzhou, 225009 Jiangsu People’s Republic of China; 2 Department of Cardiothoracic Surgery, Medical College of Yangzhou University, Yangzhou University, Yangzhou, 225009 Jiangsu People’s Republic of China; 3 Department of Medical Oncology, Clinical College of Dalian Medical University, Yangzhou, 225009 Jiangsu People’s Republic of China; 4 Department of Thoracic Surgery, Affiliated Hospital of Yangzhou University, Yangzhou University, No. 368 Hanjiang Road, Yangzhou, 225009 Jiangsu People’s Republic of China

**Keywords:** C1GALT1, Radioresistance, Epithelial–mesenchymal transition, Lung adenocarcinoma

## Abstract

**Purpose:**

Core 1β1,3-galactosyltransferase 1 (C1GALT1) exhibits elevated expression in multiple cancers. The present study aimed to elucidate the clinical significance of C1GALT1 aberrant expression and its impact on radiosensitivity in lung adenocarcinoma (LUAD).

**Methods:**

The C1GALT1 expression and its clinical relevance were investigated through public databases and LUAD tissue microarray analyses. A549 and H1299 cells with either C1GALT1 knockdown or overexpression were further assessed through colony formation, gamma-H2A histone family member X immunofluorescence, 5-ethynyl-2′-deoxyuridine incorporation, and flow cytometry assays. Bioinformatics analysis was used to explore single cell sequencing data, revealing the influence of C1GALT1 on cancer-associated cellular states. Vimentin, N-cadherin, and E-cadherin protein levels were measured through western blotting.

**Results:**

The expression of C1GALT1 was significantly higher in LUAD tissues than in adjacent non-tumor tissues both at mRNA and protein level. High expression of C1GALT1 was correlated with lymph node metastasis, advanced T stage, and poor survival, and was an independent risk factor for overall survival. Radiation notably upregulated C1GALT1 expression in A549 and H1299 cells, while radiosensitivity was increased following C1GALT1 knockdown and decreased following overexpression. Experiment results showed that overexpression of C1GALT1 conferred radioresistance, promoting DNA repair, cell proliferation, and G_2_/M phase arrest, while inhibiting apoptosis and decreasing E-cadherin expression, alongside upregulating vimentin and N-cadherin in A549 and H1299 cells. Conversely, C1GALT1 knockdown had opposing effects.

**Conclusion:**

Elevated C1GALT1 expression in LUAD is associated with an unfavorable prognosis and contributes to increased radioresistance potentially by affecting DNA repair, cell proliferation, cell cycle regulation, and epithelial–mesenchymal transition (EMT).

## Introduction

Lung cancer stands as the most prevalent cancer type and remains the foremost contributor to cancer-related fatalities worldwide. It was estimated that 2.2 million new patients were diagnosed with lung cancer and 1.8 million deaths died of lung cancer according to the GLOBOCAN 2020 (Sung et al. 2021). Lung cancer can be classified into four major histological types, lung adenocarcinoma (LUAD), lung squamous cell carcinoma, large cell carcinoma, and small cell carcinoma. Among all the histological types of lung cancer, LUAD has emerged as the dominant pathological subtype, representing >40% of lung cancer cases in numerous countries (Lortet-Tieulent et al. 2014; Meza et al. 2015; Denton et al. 2016; Shi et al. 2019; Dong et al. 2021a). Treatment strategies for lung cancer include surgery, chemotherapy, radiotherapy, target therapy, and immunotherapy. However, a substantial portion of patients experience recurrence or metastasis following comprehensive treatment, with a bleak prognosis. In fact, among individuals diagnosed with lung cancer between 2010 and 2014, the 5-year survival rate ranges from 10 to 20% in most nations (Allemani et al. 2018).

Currently, radiotherapy occupies a prominent role as one of the primary treatment modalities, applicable across all stages of lung cancer, serving both curative and palliative purposes (Bezjak et al. 2015; Brown et al. 2019). In the case of early-stage disease, stereotactic ablative radiotherapy has emerged as a standard treatment option for patients who are not suitable candidates for surgery (Vansteenkiste et al. 2013). Concurrent chemoradiotherapy stands as the standard of care for patients with inoperable locally advanced lesions (Auperin et al. 2010). Furthermore, accumulating evidence also shows the survival benefits of radiotherapy for asymptomatic or limited metastatic cases (Palma et al. 2019). Notably, palliative radiotherapy is also deployed to enhance the quality of life for individuals with incurable lung cancer (Stevens et al. 2015). However, a subset of tumor cells manages to evade the antitumor effects of radiotherapy, resulting in the emergence of a more aggressive cancer phenotype. This phenomenon curtails the efficacy of subsequent treatments (Carlos-Reyes et al. 2021). Thus, it becomes imperative to unravel the molecular mechanisms underpinning the intrinsic or acquired radioresistance in lung cancer, which will provide potential therapeutic targets for the effective management of lung cancer.

Core 1 β1, 3-galactosyltransferase 1 (C1GALT1), which is also called T-synthase, is an important enzyme responsible for transferring galactose (Gal) from UDP-galactose to GalNAcα1-Ser/Thr structure (Thomsen-Nouveau antigen, Tn antigen), facilitating the synthesis of core 1 *O*-glycan structure (Galβ1-3GalNAcα1-Ser/Thr, T antigen) which is the main modification of the Tn antigen(Ju et al. 2002a; b; Lin et al. 2018). This enzymatic process plays a critical role in GalNAc-type *O*-glycosylation (Tarp and Clausen 2008). The process of *O*-glycosylation begins with the formation of the Tn antigen catalyzed by GalNAc aminotransferases peptide (Ju and Cummings 2002; Ju et al. 2011). Besides core 1 *O*-glycan structure, Tn antigen can also be catalyzed by β1,3-*N*-acetylglucosaminyltransferase 6 to form core 3 structure (Tran and Ten 2013). Subsequently, core 1 and core 3 structures are further modified by β1,6-*N*-acetylglucosaminyltransferase to form core 2 and core 4 structures, respectively. These core structures are further extended by Gal and GlcNAc and often terminated by sulfate groups, sialic acid, GalNAc, and/or Fuc, resulting in the formation of *O*-glycans that can vary significantly in different cells despite sharing the same protein sequence (Arike and Hansson 2016). *O*-Glycosylation is often referred to as mucin-type glycosylation which is critical for mucin function as more than 80% of the mass of mucins consists of *O*-glycans (Arike and Hansson 2016). The aberrant expressions of C1GALT1, mucins, and truncated core 1 based structures (T antigen and Tn antigen) are commonly observed in a variety of human cancers (Fu et al. 2016; Hanson and Hollingsworth 2016; Jiang et al. 2018a, b; Xia et al. 2022).

C1GALT1 has important functions in oncogenesis and participates in a range of pathological processes (Sun et al. 2021; Xia et al. 2022) in a tissue-specific manner. Mice models with specific knockout of C1GALT1 promote spontaneous tumors development (Bergstrom et al. 2016; Chugh et al. 2018; Liu et al. 2020). In pancreatic ductal adenocarcinoma (PDAC), neuroblastoma, and endometrial cancer, reduced C1GALT1 expression was associated with more aggressive phenotype of tumor (Chugh et al. 2018; Lin et al. 2022; Montero-Calle et al. 2023). On the contrary, more studies have shown that elevated expression of C1GALT1 has been observed in a variety of malignant tumors, including esophageal cancer (Wang et al. 2018), gastric cancer (Dong et al. 2021a), colon cancer (Hung et al. 2014a, b), hepatocellular carcinoma (Wu et al. 2013), breast cancer (Chou et al. 2015), prostate cancer (Tzeng et al. 2018), head and neck cancer (Lin et al. 2018), PDAC (Kuo et al. 2021), and ovarian cancer (Chou et al. 2017). This upregulation of C1GALT1 is associated with the promotion of various malignant cellular phenotypes, such as enhanced cell adhesion, proliferation, migration, invasion, and treatment sensitivity, along with a reduction in immune response and surveillance, ultimately leading to a poor prognosis for cancer patients through multifactorial mechanisms (Xia et al. 2022; Wan et al. 2023). Furthermore, two reports showed that C1GALT1 could induce radioresistance in human esophageal cancer and laryngeal cancer cells through the modification of β1-integrin glycosylation (Dong et al. 2018; Zhang et al. 2018).

A recent study has shown that elevated C1GALT1 expression is associated with poor prognosis and contributes to cancer cell proliferation, migration, and invasion through upregulating RAC1 in LUAD (Dong et al. 2021b). However, whether C1GALT1 is involved in regulating radiosensitivity in LUAD remains unknown. We hypothesized that the overexpression of C1GALT1, identified as a negative indicator for patients’ prognosis in most cancer types, may alter tumor radiosensitivity in LUAD, thereby having critical consequences in cancer progression. The present study aimed to assess the clinical significance of C1GALT1 in LUAD using a combination of public databases and clinical tumor samples and found that high C1GALT1 expression in LUAD tissues was associated with lymph node metastasis and poor prognosis. Single-cell data enrichment analysis strongly suggested that C1GALT1 expression correlated with epithelial–mesenchymal transition (EMT) in LUAD. The present investigation revealed that radiation exposure could induce the upregulation of C1GALT1, N-cadherin and vimentin but reduce the expression of E-cadherin in A549 and H1299 cells. Furthermore, we confirmed that C1GALT1 could induce the radioresistance of A549 and H1299 cells by promoting DNA repair, increasing cell proliferation, inducing G_2_/M phase arrest, activating EMT, and inhibiting apoptosis, suggesting that C1GALT1 is a novel regulator of radiosensitivity in LUAD.

## Materials and methods

### Public database and bioinformatics analysis

Gene expression data and clinical information of patients with LUAD were collected from The Cancer Genome Atlas (TCGA) database (available at https://www.cancer.gov/tcga), and the Gene Expression Omnibus (GEO) database (available at https://www.ncbi.nlm.nih.gov/geo/). A total of seven GEO databases (GSE85716, GSE7670, GSE85841, GSE130779, GSE146460, GSE115002, and GSE176348) were utilized to analyze the expression of C1GALT1. The RNA-seq data from TCGA were analyzed using the EdgeR package (version 3.38) in the R program (version 3.6.3) (Robinson et al. 2010). Data were downloaded from the GEO database and values of different gene expressions were log_2_ transformed and normalized through quantile normalization for individual GEO databases. GEO databases from different platforms were pretreated with batch correction and normalization. Differential expression analysis was conducted using the limma package (version 3.22) in the R program (Ritchie et al. 2015). GSE68465 and TCGA data were further used for survival analysis.

In addition, single-cell gene expression profiles of tumor cell-enriched patient-derived xenograft cells (LC-PT-45, *n* = 34 and LC-Pt-45-Re, *n* = 43) from patients with LUAD were downloaded from GSE69405. The most significant genes correlated with C1GALT1 were used for enrichment analysis, including Gene Ontology (GO) molecular functions, Kyoto Encyclopedia of Genes and Genomes (KEGG) pathway, and hallmark enrichment, performed using Metascape (https://metascape.org/). The correlation between C1GALT1 expression and scores of 14 functional states (including stemness, invasion, metastasis, proliferation, EMT, angiogenesis, apoptosis, cell cycle, differentiation, DNA damage, DNA repair, hypoxia, inflammation, and quiescence) was further analyzed using Cancer Single-cell State Atlas (CancerSEA) database (http://biocc.hrbmu.edu.cn/CancerSEA/).

### Tissue microarray

The formalin-fixed and paraffin-embedded LUAD tissue microarray (HLugA180Su07) was obtained from Shanghai Outdo Biotech Co., Ltd. This tissue array comprises 82 pairs of LUAD tissues and their adjacent normal lung tissues, along with an additional 16 LUAD tissues. The specimens were collected between July 2004 and June 2009, with the last follow-up visit performed in August 2014. Survival information and clinical records were reviewed.

### Immunohistochemical (IHC) staining

IHC staining was performed using an immunohistochemistry kit (Beijing Zhongshan Golden Bridge Biotechnology Co., Ltd.) following the manufacturer’s instructions. A rabbit polyclonal primary antibody against C1GALT1 (HPA011294; Atlas Antibodies) was employed. After titrated the dilution, we used an antibody dilution of 1:100 for IHC. Images were captured using Aperio Digital Pathology Slide Scanners (Leica Microsystems, Inc.). The IHC staining evaluation of C1GALT1 was conducted independently by two pathologists, and the stain intensity was scored as follows: 0 (negative); 1 (weak); 2 (moderate); and 3 (strong). High expression of C1GALT1 was defined as a score of 3 in IHC staining, while low expression of C1GALT1 was defined as a score of <3 in IHC staining.

### Cell lines and cell culture

The human lung cancer cell lines A549 and H1299 were purchased from Procell Life Science & Technology Co., Ltd (Wuhan, China) and cultured in DMEM (Gibco; Thermo Fisher Scientific, Inc.) supplemented with 10% fetal bovine serum (Hyclone; Cytiva) at 37 °C in a 5% CO_2_ atmosphere.

### Transfection and plasmid constructs

The human full-length C1GALT1-overexpressing commercial and empty plasmids were purchased from Shanghai Genechem Co., Ltd. Short hairpin (sh)RNAs targeting C1GALT1, along with a scramble shRNA plasmid serving as a negative control, were designed and obtained from Shanghai Genechem Co., Ltd. The sequences for the shRNAs were as follows: shRNA#1, 5′-GCGTTGTAACAAAGTGTTGTT-3′; shRNA#2, 5′-CCTACCTTAC CTGAACGTATA-3′; and shRNA#3, 5′-GCCTTATGTAAAGCAGGGCTA-3′. The scramble sequence of shRNA was 5′-TTCTCCGAACGTGTCACGT-3′. Cell transfection was carried out using Lipo8000 Transfection Reagent (Beyotime Institute of Biotechnology) following the manufacturer’s instructions.

### Clonogenic formation assays

At 24 h after transfection with various plasmids, A549 and H1299 cells were digested using trypsin and plated into 12-well plates at a density of 200 cells/well. After an overnight culturing, the cells were exposed to a radiation dose of 2 Gy using a medical linear accelerator (Varian Medical Systems, Inc.) at a rate of 2 Gy/min. After 14 days of irradiation, the cells were fixed using 100% methanol and subsequently stained with 0.1% crystal violet (Sigma-Aldrich; Merck KGaA). The number of colonies containing >50 cells was then counted and recorded.

### Cell cycle analysis

A549 and H1299 cells were initially seeded in six-well plates at a density of 1 × 10^6^ cells/well. Subsequently, cells were transfected with various plasmids. After 24 h of transfection, the cells were exposed to a radiation dose of 2 Gy and then cultured for an additional 24 h before being harvested. For cell cycle analysis, the cells were fixed using 80% ice-cold ethanol and stained with PI/RNase staining buffer solution (BD Biosciences). Data were acquired using DxFLEX flow cytometry (Berkman Coulter, Inc.) and analyzed using ModFit LT software (Version 4.0, Verity Software House, Inc.).

### Apoptosis analysis

Cell apoptosis was analyzed using the Annexin V-FITC/PI apoptosis detection kit (A211; Vazyme Biotechnology Co., Ltd). Briefly, A549 and H1299 cells including their supernatants were collected 48 h after transfection and centrifuged followed by washing three times with PBS. Then the cells were stained using apoptosis detection kit according to the manufacturer’s instructions. Cell apoptosis was analyzed using DxFLEX flow cytometry using CytExpert software (Berkman Coulter, Inc.).

Gamma-H2A histone family member X (γ-H2AX) immunofluorescence assay.

A549 or H1299 cells transfected with either overexpression or shRNA plasmids were plated on glass coverslips in a 24-well plate 24 h after transfection. After 48 h transfection, cells were irradiated at 6 Gy. After 4 h of irradiation, cells were fixed for DNA damage assay using DNA Damage Assay Kit by γ-H2AX immunofluorescence (Beyotime Institute of Biotechnology) according to the manufacturer’s instruction. Images were captured using a fluorescence microscope at 400x. The ratio of cells with >10 γ-H2AX foci (red fluorescence) was then calculated.

### 5-Ethynyl-2′-deoxyuridine (EdU) assay

BeyoClick™ EdU-594 cell proliferation detection kit (Beyotime Institute of Biotechnology) was used to assess the proliferation of lung cancer cells (A549 and H1299) transfected with either overexpression or shRNA plasmids, according to the manufacturer’s protocol. Images were captured using a fluorescence microscope (Olympus Corporation) at 100x. The ratio of EdU positive cells (red fluorescence) to Hoechst33342 positive cells (blue fluorescence) per well was further analyzed.

### Western blot

Total protein was extracted from cells 72 h after transfection or 48 h after different doses irradiation using RIPA lysis buffer (Beyotime Institute of Biotechnology). Protein concentrations were determined using an enhanced BCA protein assay kit (Beyotime Institute of Biotechnology), and a total of 20 μg protein was loaded onto 10% SDS-PAGE gels. The separated proteins were then transferred onto 0.2 µm PVDF membranes (Roche Applied Science). Following blocking with 5% skimmed milk for 1 h at room temperature, the membranes were incubated with primary antibodies overnight at 4 °C. The primary antibodies used in the present study included C1GALT1 (1:5000; ab237734; Abcam), E-cadherin (1:5000; ab40772; Abcam), N-cadherin (1:5000; ab76011; Abcam), vimentin (1:5000, ab92547; Abcam), and GADPH (1:5000, ab9485; Abcam). Subsequently, the membranes were incubated with anti-rabbit secondary antibody (1:20,000; 7074P2; Cell Signaling Technology) conjugated with horseradish peroxidase for 1 h at room temperature. Protein bands were detected using Pierce ECL reagents (Thermo Fisher Scientific, Inc.) and visualized with a Tanon-5200 imaging system (Tanon Science and Technology Co., Ltd.).

### Statistical analysis

Data analysis was performed using SPSS 13.0 (SPSS, Inc.) statistical software, and Kaplan–Meier overall survival curves were generated using GraphPad Prism (version 8.3). Wilcoxon rank sum test was used to determine the difference of C1GALT1 IHC scores between cancer and adjacent tissues. The data are presented as means ± standard deviation (SD) and were analyzed through Student’s *t* test. Overall survival difference according to C1GALT1 expression was determined using log-rank test. Multivariable Cox proportional hazards mode analysis was used to identify predictors of overall survival. *P* < 0.05 was considered to indicate a statistically significant difference. All experiments were performed with technical triplicates.

## Results

### C1GALT1 is overexpressed in LUAD

**Fig. 1:**
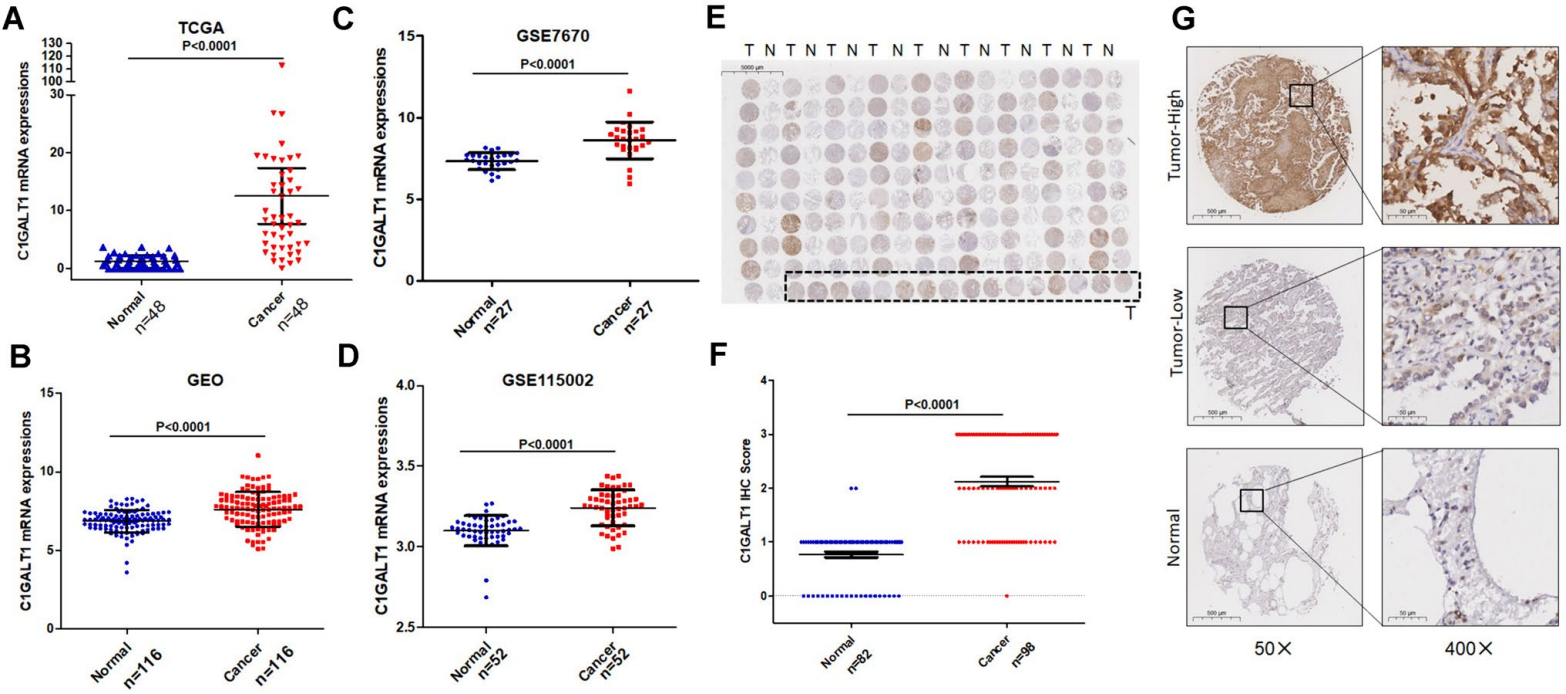
C1GALT1 is overexpressed in LUAD compared with normal tissues. **A** Analysis of C1GALT1 mRNA expression in paired cancer and normal tissues using The Cancer Genome Atlas database. **B** C1GALT1 mRNA expression in unpaired cancer and normal tissues from seven Gene Expression Omnibus databases (GSE85716, GSE7670, GSE85841, GSE130779, GSE146460, GSE115002 and GSE176348). **C**,**D** Individual Gene Expression Omnibus database (GSE7670 and GSE115002) analysis for C1GALT1 mRNA expression. **E** Immunohistochemistry images depicting C1GALT1 protein expression in LUAD tissue microarray (×5 magnification). The tissue array within the dashed box represents tumor tissue. **F** The difference in C1GALT1 immunohistochemistry scores between LUAD tissues and adjacent non-tumor tissues was assessed from tissue microarray. **G** Representative images show samples stained with C1GALT1 antibody. The magnification ratio is ×50 on the left side and ×400 on the right side. T, tumor tissue; N, normal tissue; LUAD, lung adenocarcinoma; C1GALT1, core 1β1,3-galactosyltransferase 1

To comprehensively assess the expression of C1GALT1 in LUAD, the analysis of C1GALT1 mRNA levels utilizing data from the TCGA database was initially performed. The present results demonstrated a significant increase in C1GALT1 mRNA expression in LUAD compared with that in normal lung tissues (Fig. 1A). Furthermore, we corroborated these results through an examination of data from seven GEO databases and confirmed the marked overexpression of C1GALT1 mRNA in LUAD tumors in comparison to normal tissues (Fig. 1B). Given the diversity in platforms among these seven GEO databases, a more granular approach was taken by separately analyzing data from two individual GEO databases (GSE7670 and GSE115002). The present analysis once again confirmed that C1GALT1 was highly expressed in LUAD (Fig. 1C and D). To further validate these observations, the C1GALT1 protein levels in clinical tissues were evaluated using IHC staining. The results of this analysis consistently demonstrated significantly elevated expressions of C1GALT1 protein within LUAD tissues when compared with that in adjacent non-tumor tissues (Fig. 1E–G).

### High expression of C1GALT1 is correlated with aggressive behavior and poor prognosis in LUAD

**Fig. 2:**
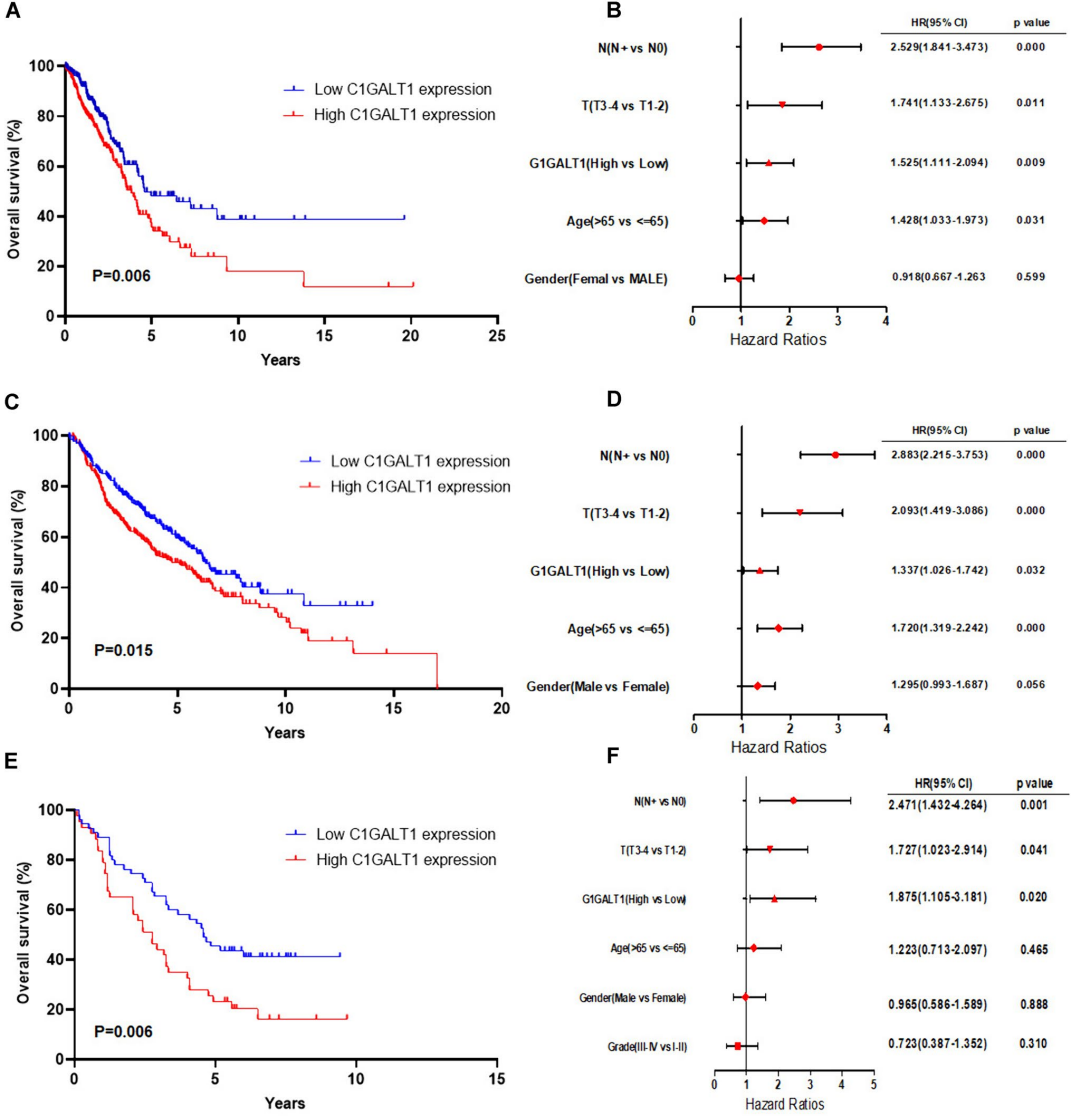
High C1GALT1 expression is correlated with poor prognosis of patients with LUAD and is an independent risk factor for overall survival. Kaplan–Meier curves for OS in patients with LUAD with different expressions of C1GALT1 from **A** TCGA database, **C** GSE68465, and **E** tissue microarray. Multivariate Cox proportional hazards models for predictors of OS in patients with LUAD from **B** TCGA database, **D** GSE68465, and **F** tissue microarray. OS, overall survival; TCGA, The Cancer Genome Atlas; LUAD, lung adenocarcinoma; C1GALT1, core 1β1,3-galactosyltransferase 1

Since C1GALT1 was overexpressed in LUAD, the association between C1GALT1 expression and clinical parameters was further analyzed. The analysis of both TCGA and GEO databases consistently revealed that high C1GALT1 expression was notably and significantly linked to shortened overall survival (Fig. 2A and C). In addition, multivariate Cox regression analysis showed that high C1GALT1 expression was an independent risk factor for overall survival across both TCGA and GEO databases (Fig. 2B and D). Next, IHC analysis was further used to confirm the results from TCGA and GEO databases. The results from tissue microarray analysis demonstrated that high C1GALT1 expression was significantly associated with lymph node metastasis and advanced T stage (Table 1). Kaplan–Meier survival analysis again revealed that patients exhibiting high expression of C1GALT1 had a poorer prognosis when compared with those with low C1GALT1 expression (Fig. 2E). Moreover, C1GALT1 was an independent risk factor for overall survival in patients with LUAD (Fig. 2F). Collectively, these comprehensive findings indicated that C1GALT1 was an important factor during the development and progression of patients with LUAD.

**Table 1:** Correlation between C1GALT1 expression and multiple clinicopathological characteristics in LUAD

Characteristics	*n*	C1GALT1 expression		*x*^2^ value	*P* value
		Low	High		
Gender					
Male	55	29	26	0.587	0.444
Female	43	26	17		
Age					
≤65	70	38	32	0.336	0.562
>65	28	17	11		
Grade					
I–II	71	43	28	2.064	0.151
III	27	12	15		
T stage^a^					
T1–2	64	41	23	4.168	0.041
T3–4	31	13	18		
Lymph node^b^					
Negative	44	30	14	4.323	0.038
Positive	53	25	28		

^a^ 3 cases missing

^b^ 1 case missing

### C1GALT1 induces the radioresistance in A549 and H1299 cells

**Fig. 3:**
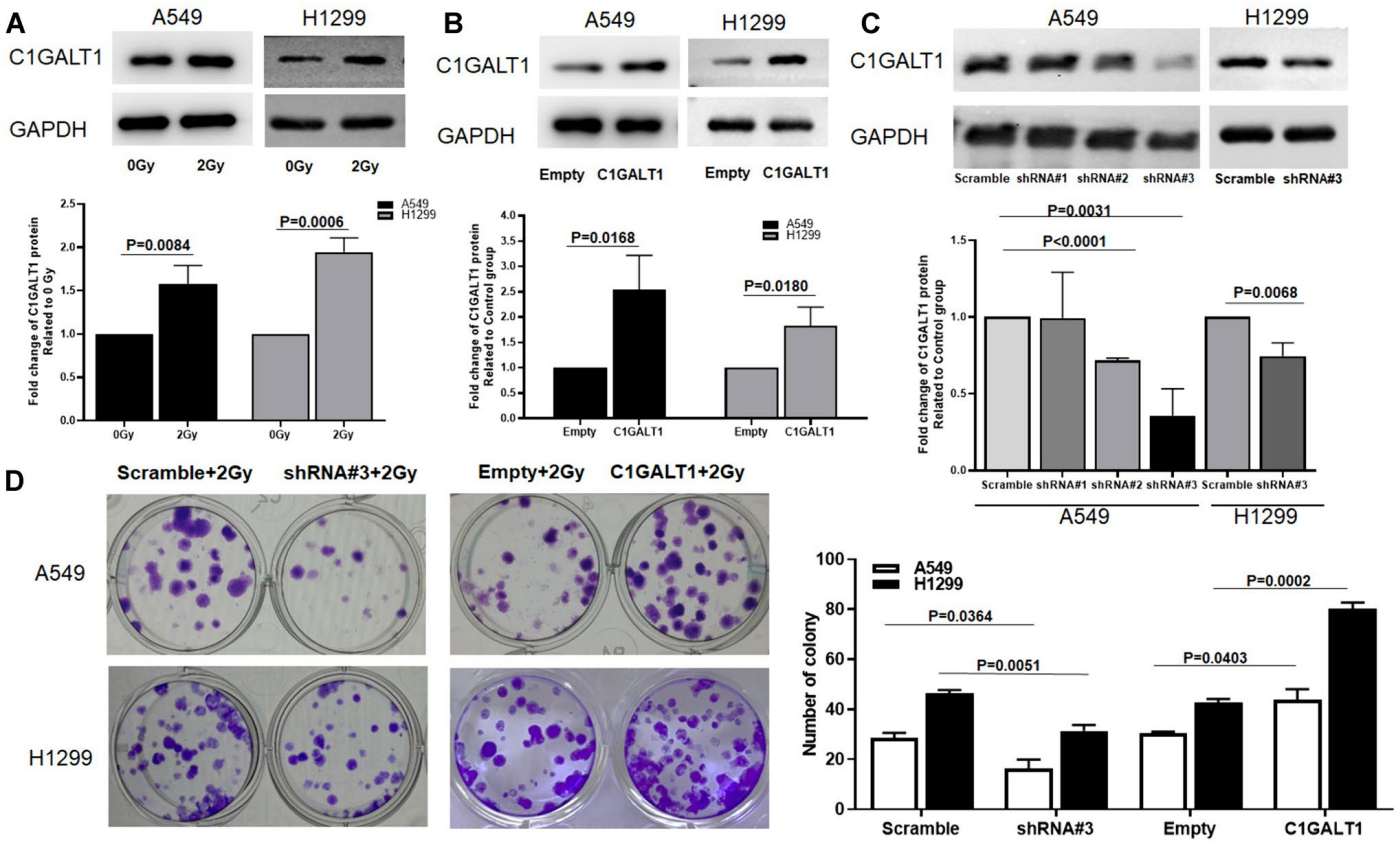
C1GALT1 regulates the radiosensitivity of A549 and H1299 cells. The empty plasmid and scramble shRNA plasmid were utilized as negative controls for the overexpression plasmid and shRNA plasmid, respectively. **A** Protein expressions of C1GALT1 were measured using western blot in A549 and H1299 cancer cells 48 h after irradiation. **B** Protein levels of C1GALT1 were upregulated in A549 and H1299 cells transfected with C1GALT1-overexpressed plasmid. **C** Knockdown of endogenous C1GALT1 using shRNA#3 exhibited the most effective C1GALT1 inhibition in A549 cells and is validated in H1299 cells. **D** Radiosensitivity was detected through colony formation experiments in A549 and H1299 cells transfected with either C1GALT1 shRNA or C1GALT1-overexpressed plasmid. shRNA, short hairpin RNA; C1GALT1, core 1β1,3-galactosyltransferase 1

Because radiotherapy is an important option for the treatment of lung cancer, the present study further explored the relationship between the C1GALT1 expression and radiosensitivity in A549 and H1299 cells. A549 and H1299 cell lines were subjected to 2 Gy irradiated, and total protein was extracted 48 h post-treatment to be used for western blot analysis. The present data showed a substantial upregulation of C1GALT1 protein levels in response to radiation exposure when compared with non-irradiated cells (Fig. 3A). Subsequently, gain- and loss-of-function experiments were performed to investigate whether C1GALT1 played a direct role in modulating the cellular response to radiation. Through western blot assays, the significant upregulation of C1GALT1 protein levels was confirmed in both A549 and H1299 cells transfected with a C1GALT1-overexpressing plasmid (Fig. 3B). In addition, to establish the efficacy of C1GALT1 inhibition, the shRNA#3 was used since it exhibited the most effective inhibition of C1GALT1 in A549 cells to knock down endogenous C1GALT1 and was also validated in H1299 cells (Fig. 3C). After 2 weeks of irradiation at 2 Gy, colony formation experiments showed that the number of colonies formed by A549 and H1299 cells transfected with C1GALT1 shRNA was significantly reduced compared with those transfected with scramble shRNA plasmid (Fig. 3D). Conversely, the number of colonies formed by A549 and H1299 cells transfected with C1GALT1-overexpressed plasmid exhibited a substantial increase compared with the empty plasmid. (Fig. 3D).

### C1GALT1 reduces DNA damage in A549 and H1299 cells

**Fig. 4:**
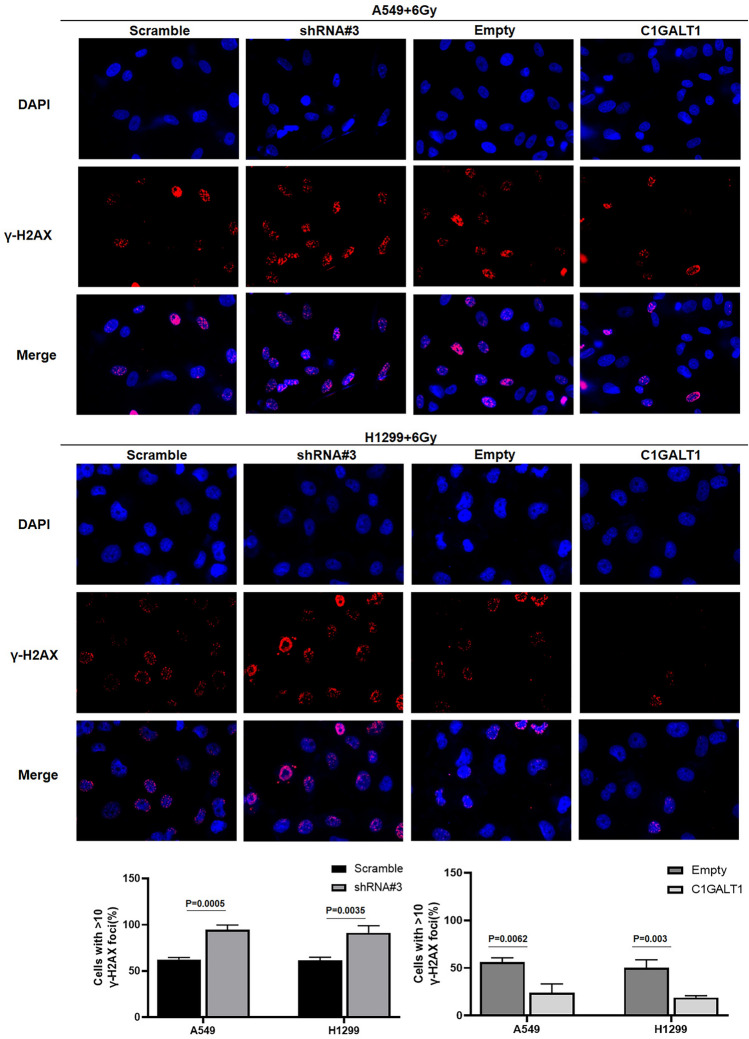
The influence of C1GALT1 on radiation-induced DNA damage in A549 and H1299 cells. A549 and H1299 cancer cells transfected with indicated plasmids were exposed to radiation with 6 Gy. At 4 h after irradiation, A549 and H1299 cells were stained with γ-H2AX. The ratio of cells with >10 γ-H2AX foci (red fluorescence) is shown. The empty plasmid and scramble shRNA plasmid were utilized as negative controls for the overexpression plasmid and shRNA plasmid, respectively. The magnification ratio is ×400. C1GALT1, core 1β1,3-galactosyltransferase 1; γ-H2AX, gamma-H2A histone family member X

To elucidate the role of C1GALT1 in the DNA damage response of lung cancer cells, we quantified the number of γ-H2AX foci post-irradiation. Notably, C1GALT1 overexpression resulted in a significant reduction in the number of γ-H2AX foci following 6 Gy irradiation when compared with cells transfected with empty plasmid (Fig. 4). Conversely, C1GALT1 inhibition led to a substantial increase in the number of γ-H2AX foci post-irradiation. These results suggest that C1GALT1 is closely correlated with the radiosensitivity of A549 and H1299 cells, possibly by promoting the more efficient repair of DNA damage.

### Bioinformatics analysis of single cell sequencing data and cancer-associated cellular status regulated by C1GALT1

**Fig. 5:**
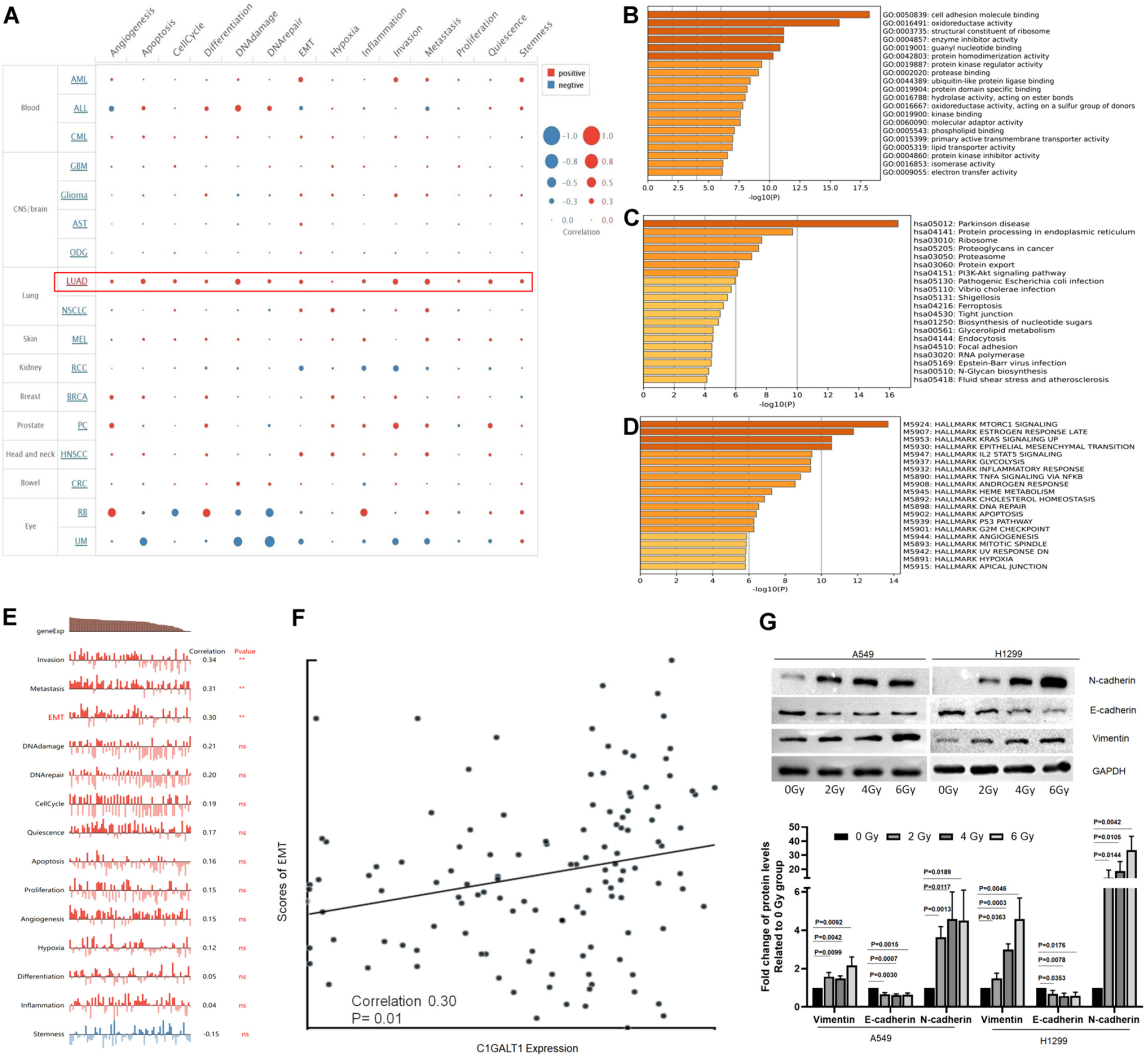
Bioinformatics analysis of single cell sequencing data and cancer-associated cellular status regulated by C1GALT1 and expression changes of EMT markers induced by irradiation in cancer cells. **A** The correlation between the C1GALT1 expression and 14 cancer-associated functional states in different cancer types, using single-cell sequencing datasets in the Cancer Single-cell State Atlas (http://biocc.hrbmu.edu.cn/CancerSEA/) database. **B** Gene Ontology enrichment (molecular functions) analysis of most significantly correlated genes for C1GALT1 using Metascape (https://metascape.org/). **C** Kyoto Encyclopedia of Genes and Genomes pathway enrichment analysis of most significantly correlated genes for C1GALT1 using Metascape. **D** Hallmark enrichment analysis of most significantly correlated genes for C1GALT1 using Metascape. **E** Correlations between 14 cellular status and C1GALT1 expression of single cell (LC-PT-45, *n* = 34 and LC-Pt-45-Re, *n* = 43) from patients with LUAD. **F** Scatter diagram of EMT scores and C1GALT1 expression. **G** Protein levels of N-cadherin, E-cadherin, and vimentin in A549 and H1299 cells exposure to 0, 2, 4, 6 Gy irradiation. ** *P* < 0.01. Ns, not significant; EMT, epithelial–mesenchymal transition; C1GALT1, core 1β1,3-galactosyltransferase 1; LUAD, lung adenocarcinoma

To further explore the underlying mechanisms by which C1GALT1 regulates radiosensitivity in LUAD, bioinformatics analysis was conducted using single-cell sequencing data (CancerSEA database) and its impact on cancer-related functional states was explored at the single cell level. Pan-cancer analysis revealed that C1GALT1 exhibited a positive correlation with most of functional states in LUAD (Fig. 5A). To further elucidate the potential regulatory mechanisms governed by C1GALT1 in LUAD, genes with *P* < 0.01 for the spearman’s rank correlations with C1GALT1 were acquired for GO, KEGG pathway, and hallmark enrichment analysis based on the single-cell gene expression profiles of LC-PT-45(*n* = 34) and LC-Pt-45-Re (*n* = 43) cells. In the GO (molecular functions) analysis, the present findings revealed that C1GALT1 was associated with various biological processes, such as cell adhesion molecule binding, oxidoreductase activity, structural constituent of ribosome, enzyme inhibitor activity, guanyl nucleotide binding, and protein homodimerization activity (Fig. 5B). Furthermore, the KEGG pathway analysis showed potential correlations between C1GALT1 and pathways including Parkinson’s disease, protein processing, ribosome, proteoglycans in cancer, proteasome, protein export and PI3K/Akt signaling pathway (Fig. 5C). The hallmark analysis highlighted associations between C1GALT1 and various biological processes, including mTORC1 signaling, estrogen response late, KRAS signaling, EMT, IL2/STAT5 signaling, glycolysis, inflammatory response, TNF-ɑ signaling via NF-ĸB, androgen response, heme metabolism, cholesterol homeostasis, DNA repair, apoptosis, p53 pathway, G_2_/M checkpoint, and angiogenesis (Fig. 5D). Cellular functional states analysis showed a significant correlation between C1GALT1 expression and invasive, metastatic and EMT-related processes in single cell RNA-Seq datasets (LC-PT-45 and LC-Pt-45-Re) of LUAD (Fig. 5E). Furthermore, C1GALT1 expression exhibited a positive correlation with the EMT score in LUAD (correlation = 0.3, Fig. 5F). As the hallmark gene enrichment and the Cancer Single-cell State Atlas consistently demonstrated a significant regulatory influence of C1GALT1 on the EMT which is an important biological change that plays an important role in inducing radioresistance (Hay 2005; Josson et al. 2010; Tiwari et al. 2012), the present study further investigates the expression of pivotal EMT markers (E-cadherin, N-cadherin and vimentin) in irradiated LUAD A549 and H1299 cells. The present findings indicated that the protein expression levels of vimentin and N-cadherin increased with escalating radiation doses (2, 4 and 6 Gy), while E-cadherin demonstrated a corresponding downregulation (Fig. 5G). This suggests that radiation exposure activates EMT in A549 and H1299 cells, aligning with our earlier observations from the single-cell data analysis.

### C1GALT1 regulated the cell cycle distribution of A549 and H1299 cells

**Fig. 6:**
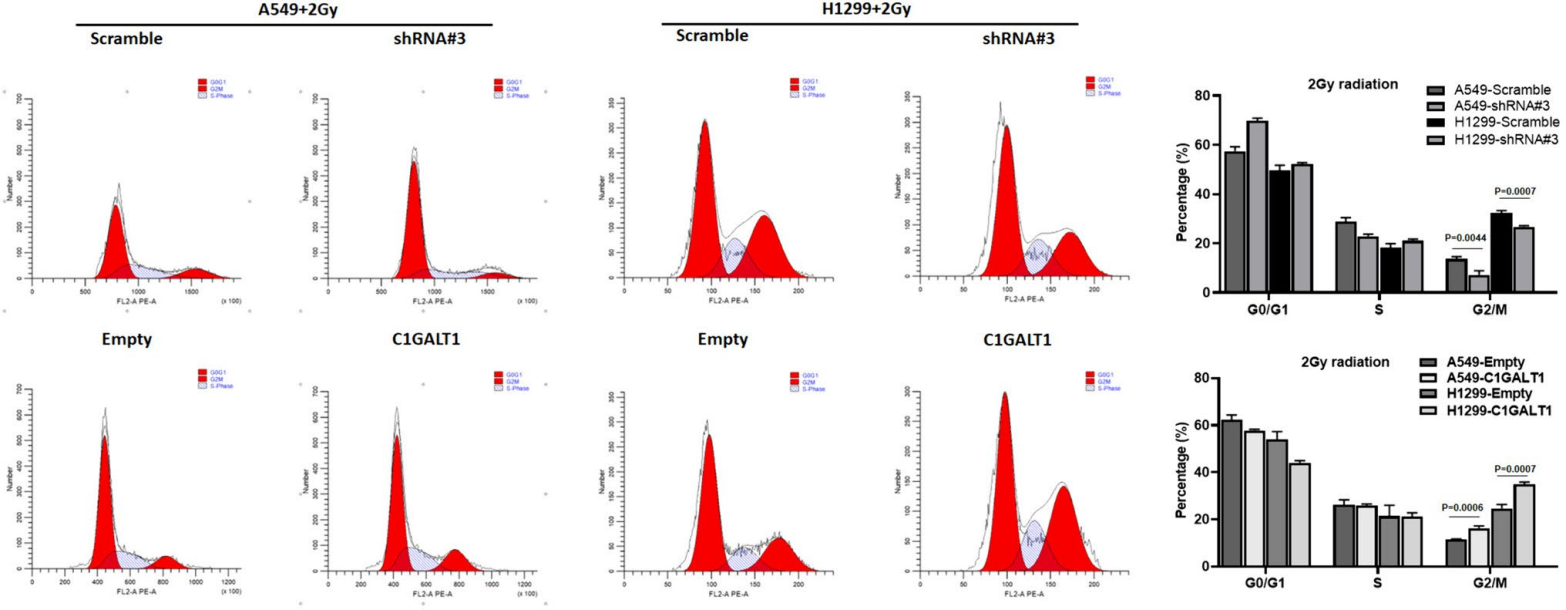
C1GALT1 regulates the radiation-induced cell cycle distribution of A549 and H1299 cells after 2 Gy irradiation. Cell cycle distribution was detected in C1GALT1 knockdown or overexpression A549 and H1299 cells 48 h after 2 Gy irradiation. The empty plasmid and scramble shRNA plasmid were utilized as negative controls for the overexpression plasmid and shRNA plasmid, respectively. Average percentage numbers of cells in each phase were calculated from triplicate experiments. C1GALT1, core 1β1,3-galactosyltransferase 1

A previous study demonstrated that changes in cell cycle distribution are closely correlated with the radiosensitivity of cancer cells (Yang et al. 2016). To support the results of hallmark analysis, the present study aimed to investigate whether C1GALT1 could influence radiation-induced cell cycle distribution in A549 and H1299 cells with either C1GALT1 knockdown or overexpression. The results demonstrated that, compared with the scramble shRNA plasmid transfected cells, C1GALT1 knockdown A549 and H1299 cells treated with 2 Gy irradiation exhibited a reduced number of cells in the G_2_/M phase (Fig. 6). Conversely, C1GALT1 overexpression cells treated with 2 Gy irradiation displayed an increased number of cells in the G_2_/M phase compared with the empty plasmid transfected cells (Fig. 6). These findings suggest that C1GALT1 promotes radiation-induced G_2_/M arrest, which might be a contributing factor to radioresistance in LUAD cells.

### C1GALT1 promotes proliferation in A549 and H1299

**Fig. 7:**
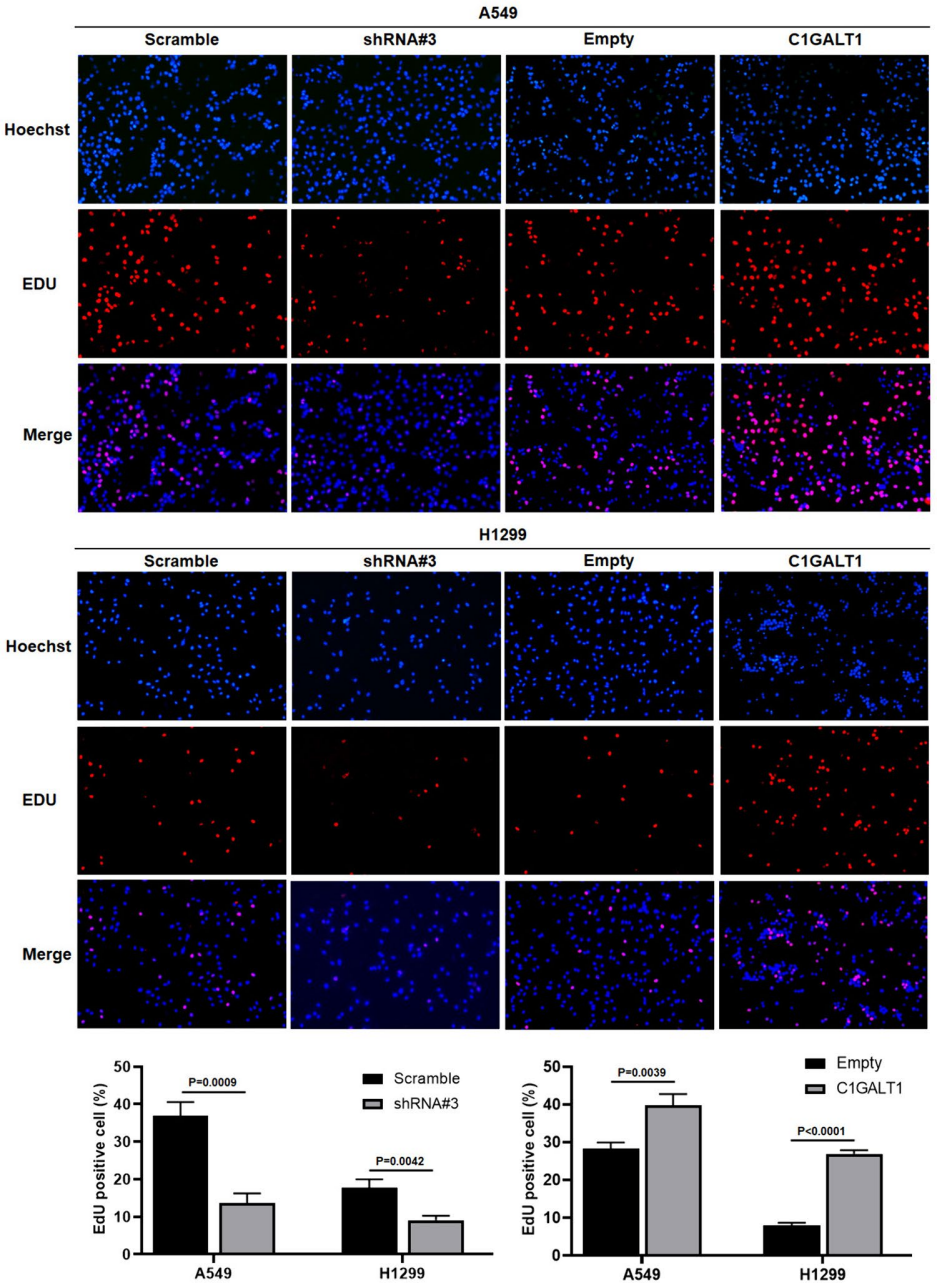
Cell proliferation is assessed using a 5-ethynyl-2′-deoxyuridine assay in A549 and H1299 cell lines after transfection with either core 1β1,3-galactosyltransferase 1-overexpressed plasmid or short hairpin RNA. The empty plasmid and scramble shRNA plasmid were utilized as negative controls for the overexpression plasmid and shRNA plasmid, respectively. The ratio of 5-ethynyl-2′-deoxyuridine positive cells (red fluorescence) to Hoechst33342 positive cells (blue fluorescence) per well was calculated. The magnification ratio is ×100

The present study delved deeper into the influence of C1GALT1 on cell proliferation using EdU assay. The present findings demonstrated that the overexpression of C1GALT1 significantly enhanced the proliferative activity of both A549 and H1299 cell lines. Conversely, the suppression of C1GALT1 expression led to a significant reduction in cell proliferation (Fig. 7).

### C1GALT1 inhibits apoptosis in A549 and H1299 cells

**Fig. 8:**
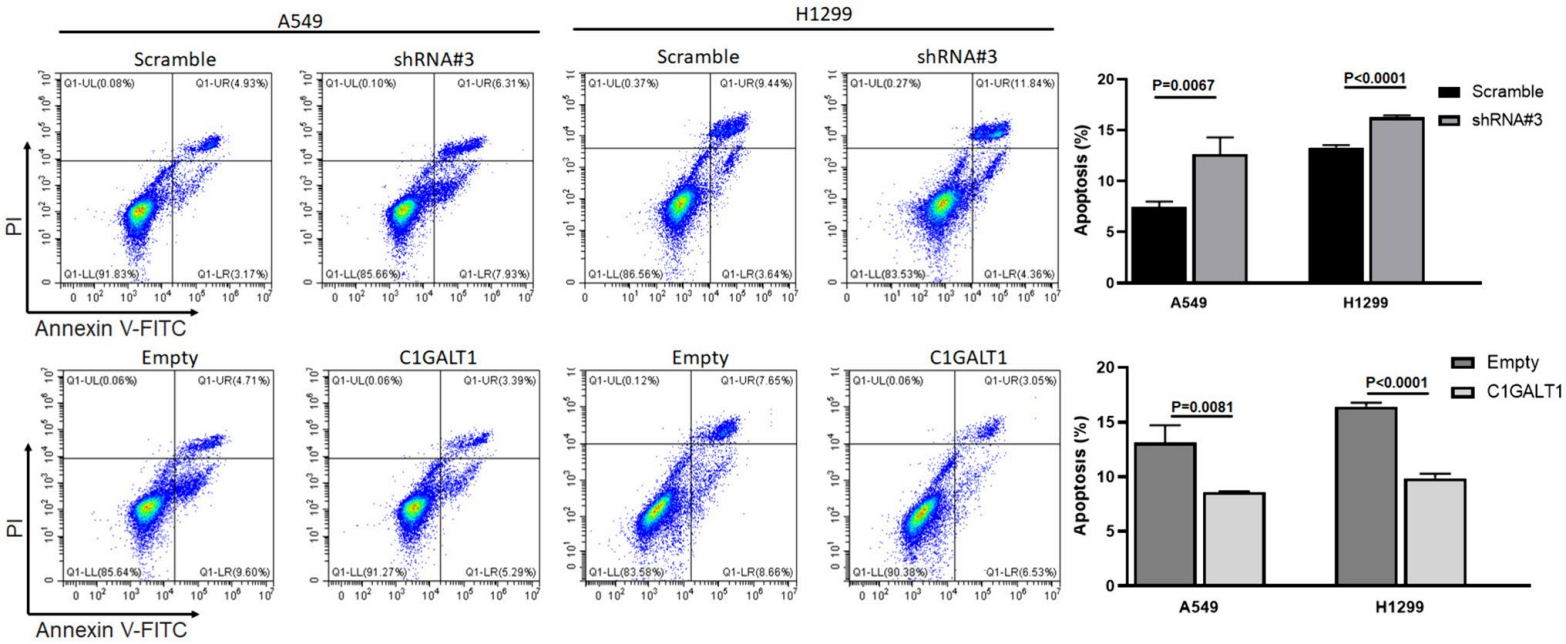
Analysis of apoptosis is assessed using flow cytometry in the A549 and H1299 cell lines after transfection with either core 1β1,3-galactosyltransferase 1-overexpressed plasmid or short hairpin RNA. The empty plasmid and scramble shRNA plasmid were utilized as negative controls for the overexpression plasmid and shRNA plasmid, respectively

The impact of C1GALT1 on apoptosis in A549 and H1299 cells was investigated using flow cytometry analysis. The present findings revealed that C1GALT1 exerted an inhibitory effect on apoptosis in A549 and H1299 cells (Fig. 8). The rate of cell apoptosis notably decreased upon C1GALT1 overexpression. By contrast, C1GALT1 inhibition led to an increased rate of cell apoptosis in both A549 and H1299 cells (Fig. 8).

### C1GALT1 regulates expressions of E-cadherin, N-cadherin, and vimentin in A549 and H1299 cells

**Fig. 9:**
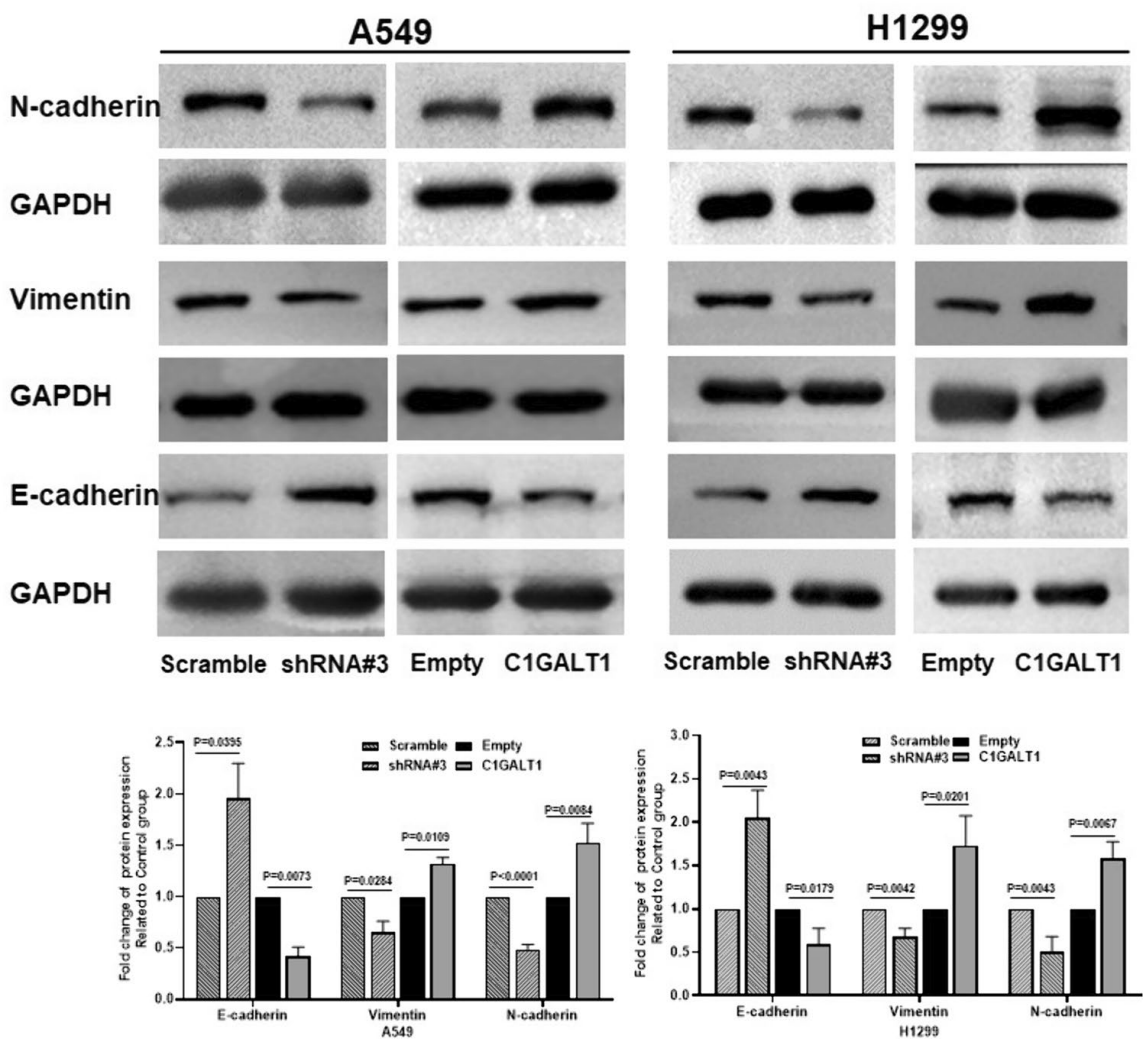
Analysis of C1GALT1 protein expressions in A549 and H1299 cell lines after transfection with C1GALT1-overexpressed plasmid or shRNA using western blot assay. C1GALT1 induces epithelial–mesenchymal transition of A549 and H1299 cells. The empty plasmid and scramble shRNA plasmid were utilized as negative controls for the overexpression plasmid and shRNA plasmid, respectively. Relative protein expressions are expressed as mean ± standard deviation. C1GALT1, core 1β1,3-galactosyltransferase 1

To explore the potential mechanisms underlying the regulation of radiosensitivity by C1GALT1 in LUAD, changes in the levels of E-cadherin, N-cadherin, and vimentin were assessed through western blot analysis. The present findings revealed that in A549 and H1299 cells transfected with a C1GALT1 overexpression plasmid, there was a significant increase in protein levels of vimentin and N-cadherin, while the protein level of E-cadherin was markedly decreased (Fig. 9). By contrast, when C1GALT1 expression was knocked down in A549 and H1299 cells, protein levels of vimentin and N-cadherin decreased significantly, while the E-cadherin expression was significantly increased compared with that in cells transfected with the scramble shRNA plasmid (Fig. 9). These results suggest that C1GALT1 is associated with the induction of EMT, indicating that C1GALT1-mediated regulation of radioresistance in LUAD may involve EMT processes.

## Discussion

The present study revealed that high expression of C1GALT1 in LUAD is associated with lymph node metastasis and poor prognosis. In addition, overexpression of C1GALT1 reduces the radiosensitivity of A549 and H1299 cells to radiation, while inhibition of C1GALT1 expression can reverse the radioresistance of these cancer cells. Mechanistically, C1GALT1 plays a protective role against radiation-induced DNA damage, promotes cell proliferation, inhibits apoptosis, induces radiation-induced G_2_/M phase arrest, upregulates vimentin and N-cadherin expressions, and downregulates E-cadherin expression. These findings collectively suggest that elevated C1GALT1 expression contributes to the radioresistance of LUAD possibly by affecting DNA repair, cell proliferation, cell cycle regulation, and EMT.

Unlike the lung squamous cell carcinoma, LUAD is recognized for its significant heterogeneity in both behavior and biological characteristics. Early in 2011, a new classification system using comprehensive histologic subtyping to define the predominant pattern (lepidic, acinar, papillary, micropapillary, or solid) for invasive LUAD was proposed by the International Association for the Study of Lung Cancer (IASLC), the American Thoracic Society (ATS), and the European Respiratory Society (ERS) (Travis et al. 2011). This classification system was then adopted by the World Health Organization (WHO) in 2015 and updated specifically for invasive nonmucinous LUAD in 2021(Nicholson et al. 2022). In nonmucinous LUAD, multiple independent cohorts have demonstrated that micropapillary/solid predominant adenocarcinomas carry a higher risk of recurrence and disease-specific death compared to those predominantly composed of lepidic/acinar/papillary structures (Yoshizawa et al. 2011, 2013; Warth et al. 2012; Gu et al. 2013; Hung et al. 2013; Hung et al. 2014a, b). Furthermore, in the eighth edition of the TNM staging system, invasive size was proposed as an alternative to total tumor size for the T descriptor of nonmucinous LUAD because utilizing invasive size might prevent the overestimation of staging that may occur when determined based on total tumor size (Yoshizawa et al. 2011; Tsutani et al. 2013; Travis et al. 2016; Kameda et al. 2018). Compared to total tumor size, employing invasive size for the T descriptor demonstrated enhanced prognostic discrimination in predicting recurrence by identifying a larger proportion of downstaged patients with improved prognosis in early-stage nonmucinous LUAD(Kameda et al. 2018). Therefore, comprehending the molecular mechanisms that provide valuable insights into the malignant phenotype of LUAD is crucial for formulating personalized treatment strategies in patients with this type of cancer.

At present, the role of C1GALT1 can be complex and context-dependent, leading to differing effects observed in various studies and cancer types (Chugh et al. 2018; Kuo et al. 2021; Lin et al. 2022). In mice models lacking intestinal epithelial core 1 *O*-glycans (IEC C1GALT1^−/−^ mice) or gastric epithelial *O*-glycans (GEC C1galt1^−/−^ mice), spontaneous colitis and gastritis, as well as gastric or colon tumors, were developed, respectively (Fu et al. 2011; Bergstrom et al. 2016; Liu et al. 2020). Pancreas-specific knockout of C1GALT1 in the Kras^G12D/+^, Trp53^R172H/+^, Pdx-1-Cre (KPC) model of PDAC promoted early tumors and metastasis (Chugh et al. 2018). These results suggested that the truncated form of *O*-glycan mediated by C1GALT1 knock out was closely associated with tumor development and progression. In PDAC, neuroblastoma, as well as endometrial cancer, the expression of C1GALT1 was significantly decreased in poorly differentiated samples compared to well-differentiated samples (Chugh et al. 2018; Lin et al. 2022; Montero-Calle et al. 2023). In addition, reduced C1GALT1 expression was associated with improved survival of neuroblastoma patients (Lin et al. 2022). However, in the study conducted by Kuo et al. (2021) with PDAC, overexpressed C1GALT1 in PDAC was associated with poor disease-free and overall survival, and C1GALT1 knockdown suppressed aggressiveness and increased gemcitabine sensitivity of PDAC cells, suggesting that the exact function of C1GALT1 in PDAC may require further investigation. In most other cancers, aberrantly high expression of C1GALT1 has been found, and it is generally considered an oncogene (Sun et al. 2021). C1GALT1 can promote malignant behaviors of cancer cells, including proliferation, migration, invasion, metastasis, tumor stem cells, radiation resistance, chemotherapeutic drug sensitivity (Wu et al. 2013; Hung et al. 2014a, b; Liu et al. 2014; Chou et al. 2015,2017; Lin et al. 2018; Dong et al. 2018; Zhang et al. 2018; Kuo et al. 2021), while also reducing immune response and surveillance (Wan et al. 2023). Wan et al. found that inhibition of C1GALT1 led to a significant reduction in cell proliferation, migration, adhesion, and the ability of colony formation in human colon cancer cells. Furthermore, inhibition of C1GALT1 caused a significant reduction of galectin-3-mediated cell–cell aggregation and cell adhesion to basement proteins, while resulting in an increase of galactose-type lectin (MGL)-mediated heterotypic aggregates formed by macrophages with cancer cells (Wan et al. 2023). Intriguingly, in mouse models with lower tumor C1GALT1 expression, more MGL-expressing macrophages and dendritic cells were attracted into the tumor surroundings (Wan et al. 2023). Recent reports also linked high C1GALT1 expression to poor prognosis and the promotion of cancer cell proliferation, migration, and invasion through the regulation of RAC1 in LUAD (Dong et al. 2021b). In the current study, 2 primary public databases (TCGA and GEO) were utilized in addition to clinical samples (98 tumor tissues) to investigate the clinical significance of C1GALT1. In alignment with previous research (Dong et al. 2021b), the present results once again reaffirmed that C1GALT1 was upregulated in LUAD tissues compared with adjacent normal tissues. Furthermore, high C1GALT1 expression was associated with aggressive behavior (higher T stage and lymph node metastasis) in LUAD and was an independent prognostic risk factor for overall survival. These findings suggested that C1GALT1 might play crucial roles in the tumorigenicity and progression of LUAD and could potentially serve as a clinical predictor of aggressiveness in LUAD patients.

Radiotherapy is a crucial component of the treatment strategy for patients with lung cancer, employed for both curative and palliative purposes. It was estimated that >60% of patients with lung cancer needed radiotherapy at some point during their disease course (Kong et al. 2014). However, the clinical benefits of radiotherapy can be limited in certain patients due to inherent or acquired radioresistance, underscoring the need to explore the underlying molecular mechanisms responsible for this resistance. Recent studies indicated that C1GALT1 functions as a regulator of radiosensitivity in human esophageal cancer and laryngeal cancer. Zhang et al. (2018) found that high expression of C1GALT1 was associated with increased resistance to radiotherapy and suppressing C1GALT1 expression enhanced the radiosensitivity of esophageal cancer cells. Furthermore, Dong et al. (2018) reported that overexpression of C1GALT1 enhanced radioresistance in radiosensitive laryngeal cells (Hep-2 min) while knocking down C1GALT1 reduced radioresistance in radioresistant laryngeal cells (Hep-2max). The present study aimed to investigate the role of C1GALT1 in modulating radiosensitivity in lung cancer cell lines. The current study demonstrated that knocking down C1GALT1 resulted in increased radiosensitivity of A549 and H1299 cells when subjected to irradiation. Conversely, the overexpression of C1GALT1 had the opposite effect, enhancing radioresistance in these lung cancer cell lines. These results suggest a close association between C1GALT1 and radioresistance in lung cancer. The precise mechanisms underlying this relationship need to be further elucidated.

There are several validated mechanisms of C1GALT1 contributing to aggressive cancer behaviors including radioresistance. Briefly, C1GALT1 could modify the *O*-glycosylation of several proteins including integrin β1, FGFR2, MET, MUC1, EGFR, and integrin α5 (Wu et al. 2013; Hung et al. 2014a, b; Chou et al. 2015; Dong et al. 2018; Lin et al. 2018; Zhang et al. 2018; Dong et al. 2021a), thereby regulating their activity and functioning as an oncogene. Importantly, suppression of C1GALT1 expression not only inhibited the development and progression of the tumor itself but also attracted more macrophages and dendritic cells to the tumor microenvironment through MGL (Wan et al. 2023). Previous mechanistic investigations in esophageal cancer and laryngeal cancer highlighted the role of C1GALT1-mediated *O*-glycosylation of β1-integrin in regulating radiosensitivity (Dong et al. 2018; Zhang et al. 2018). In the current study, an enrichment analysis of single-cell sequencing data was performed and the cancer-associated cellular processes influenced by C1GALT1 were examined through public database. The present findings revealed that C1GALT1 is associated with several cancer-related cellular processes, including DNA repair, apoptosis, EMT, and G_2_/M checkpoint regulation. These insights provide a comprehensive view of how C1GALT1 may influence cancer behavior and radioresistance.

The present study demonstrated that C1GALT1 plays a multifaceted role in radioresistance. Specifically, C1GALT1 protects against radiation-induced DNA damage, promotes cell proliferation, and inhibits apoptosis. Changes in cell cycle distribution were previously reported to be associated with radioresistance (Yang et al. 2016; Zhang et al. 2018). Disrupting the G_2_ checkpoint can reduce cell cycle arrest induced by irradiation, thus diminishing the radioresistance of tumor cells (Qin et al. 2014; Busch et al. 2017). The present findings support this notion, as it was observed that overexpression of C1GALT1 led to an accumulation of cells in the G_2_/M phase after irradiation. Conversely, inhibiting C1GALT1 abrogated the G_2_/M phase arrest following irradiation. These results highlight the role of C1GALT1 in promoting irradiation-induced G_2_/M phase arrest, which could contribute to radioresistance in A549 and H1299 cells.

EMT, an important biological process in which epithelial cells transition to a mesenchymal phenotype, plays crucial roles in cancer progression (Pastushenko and Blanpain 2019). During EMT, cells reduce the expression of epithelial genes, such as E-cadherin, ZO-1, and occludin, while increasing the expression of mesenchymal genes like N-cadherin, Vimentin, and fibronectin (Hernandez-Vega et al. 2020; Huang et al. 2022). This results in a distinct cellular characteristic, including heightened stemness, enhanced invasiveness, increased drug resistance, and the ability to form metastases (Zhang and Weinberg 2018). In addition, accumulating evidence highlighted the pivotal role of EMT as a crucial inducer of radioresistance in cancer cells (Theys et al. 2011; Nantajit et al. 2015; Zhou et al. 2020; Qiao et al. 2022). Loss of E-cadherin was shown to promote radioresistance of breast cancer MDA-MB 231 cells as determined by clone formation assay (Theys et al. 2011). Furthermore, radioresistant cancer cells often exhibit an EMT phenotype characterized by a reduction in epithelial markers (E-cadherin) and an increase in mesenchymal markers (Snail1, vimentin and N-cadherin) in non-small cell lung cancer and pancreatic cancer (Gomez-Casal et al. 2013; Jiang et al. 2018a, b). Mechanistically, the progression of EMT is regulated by intricate signaling pathways that can ultimately collaborate to induce radioresistance. Theses pathways include TGF-β pathway, Wnt pathway, Notch pathway, EGFR pathway, NF-κB pathway, PI3K/AKT pathway, ERK pathway, IL-6/STAT3 pathway (Zhou et al. 2020). Cancer stem cell (CSC) markers, such as CD44, CD29, and CD90, could also induce EMT-related radioresistance through various pathways (Zhou et al. 2020). In addition, noncoding RNAs, including microRNAs, lincRNAs, and circRNAs, can regulate radiosensitivity by inhibiting EMT (Zhou et al. 2020). In lung cancer, Tan et al. (2020) found that radiation induced EMT phenotype of cancer cell by regulating PI3K/AKT-ras-related C3 botulinum toxin substrate 1 (RAC1) pathway. RAC1, in turn, induced radioresistance of cancer cells by promoting EMT through regulating the PAK1-LIMK1-Cofilins signaling (Tan et al. 2020). Like the previous study, the present study also demonstrated that irradiation induced EMT phenotypes in A549 and H1299 cells, as evidenced by changes in the expression of EMT markers (N-cadherin, E-cadherin, and vimentin). Furthermore, the current data indicated that C1GALT1 promotes EMT phenotype in A549 and H1299 cells, while inhibition of C1GALT1 abrogates EMT phenotype of cancer cells. These findings suggest that C1GALT1-mediated radioresistance may be associated with the induction of EMT phenotype in LUAD. Recently, Dong et al. (2021a) found that C1GALT1 can activate the PI3K/Akt pathway by modifying *O*-linked glycosylation on integrin α5, promoting cell growth, and enhancing metastasis in gastric cancer. In addition, C1GALT1 also can promote cell growth and metastasis by positively regulating RAC1 in LUAD (Dong et al. 2021b). Combing previous reports (Tan et al. 2020; Dong et al. 2021a, b) with our results, we hypothesize that in LUAD, C1GALT1 may potentially exert a radioresistance effect by promoting the EMT phenotype through the PI3K/Akt-RAC1 signaling pathway in LUAD. Further investigations are warranted to elucidate the specific mechanisms by which C1GALT1 modulates radioresistance via EMT.

There are some limitations in the present study. First, while the preliminary results of our current in vitro experiments suggest that C1GALT1 plays a crucial role in promoting radioresistance in lung cancer cells, the absence of in vivo experiments is a notable limitation. Future research should incorporate animal models to validate the in vitro findings. Second, our study has confirmed that C1GALT1 could regulate DNA repair, cell proliferation, cell cycle regulation, and EMT, which are potentially associated with radioresistance in lung cancer cells. However, the precise underlying mechanisms of C1GALT1-mediated radioresistance are not fully elucidated in the present study. Future investigations can employ high-throughput analysis, in addition to bioinformatics approaches, to explore the signal regulation pathways associated with C1GALT1-mediated radioresistance. Moreover, considering the importance of EMT in radioresistance, further research on how C1GALT1 regulates EMT becomes a significant avenue for future work. Finally, it is essential to verify the role of C1GALT1-mediated protein *O*-glycosylation in the radioresistance of LUAD. Specifically, further investigation into the *O*-glycosylation of integrin β1 and integrin α5 is warranted.

In summary, the present study demonstrated the significant role of C1GALT1 in lung cancer progression and its association with poor prognosis. It also established that C1GALT1 plays a crucial role in promoting radioresistance in lung cancer cells potentially by affecting DNA repair, cell proliferation, cell cycle regulation, and EMT. These findings offer valuable insights into the mechanisms underlying radioresistance in lung cancer and may open new avenues for therapeutic interventions. Future research should explore potential mechanisms through which C1GALT1 impacts radioresistance of LUAD.

## Data Availability

The datasets used and/or analyzed during the current study are available from the corresponding author upon reasonable request.

## References

[CR1] Allemani C, Matsuda T, Di Carlo V, Harewood R, Matz M, Niksic M, Bonaventure A, Valkov M, Johnson CJ, Esteve J, Ogunbiyi OJ, Azevedo ESG, Chen WQ, Eser S, Engholm G, Stiller CA, Monnereau A, Woods RR, Visser O, Lim GH, Aitken J, Weir HK, Coleman MP (2018) Global surveillance of trends in cancer survival 2000–14 (CONCORD-3): analysis of individual records for 37 513 025 patients diagnosed with one of 18 cancers from 322 population-based registries in 71 countries. Lancet 391(10125):1023–1075. 10.1016/S0140-6736(17)33326-3 29395269 10.1016/S0140-6736(17)33326-3 PMC5879496

[CR2] Arike L, Hansson GC (2016) The densely *O*-glycosylated MUC2 mucin protects the intestine and provides food for the commensal bacteria. J Mol Biol 428(16):3221–3229. 10.1016/j.jmb.2016.02.010 26880333 10.1016/j.jmb.2016.02.010 PMC4982847

[CR3] Auperin A, Le Pechoux C, Rolland E, Curran WJ, Furuse K, Fournel P, Belderbos J, Clamon G, Ulutin HC, Paulus R, Yamanaka T, Bozonnat MC, Uitterhoeve A, Wang X, Stewart L, Arriagada R, Burdett S, Pignon JP (2010) Meta-analysis of concomitant versus sequential radiochemotherapy in locally advanced non-small-cell lung cancer. J Clin Oncol 28(13):2181–2190. 10.1200/JCO.2009.26.2543 20351327 10.1200/JCO.2009.26.2543

[CR4] Bergstrom K, Liu X, Zhao Y, Gao N, Wu Q, Song K, Cui Y, Li Y, McDaniel JM, McGee S, Chen W, Huycke MM, Houchen CW, Zenewicz LA, West CM, Chen H, Braun J, Fu J, Xia L (2016) Defective intestinal mucin-type *O*-glycosylation causes spontaneous colitis-associated cancer in mice. Gastroenterology 151(1):152–164. 10.1053/j.gastro.2016.03.039 27059389 10.1053/j.gastro.2016.03.039 PMC5068133

[CR5] Bezjak A, Temin S, Franklin G, Giaccone G, Govindan R, Johnson ML, Rimner A, Schneider BJ, Strawn J, Azzoli CG (2015) Definitive and adjuvant radiotherapy in locally advanced non-small-cell lung cancer: American Society of Clinical Oncology Clinical Practice Guideline Endorsement of the American Society for Radiation Oncology evidence-based clinical practice guideline. J Clin Oncol 33(18):2100–2105. 10.1200/JCO.2014.59.2360 25944914 10.1200/JCO.2014.59.2360

[CR6] Brown S, Banfill K, Aznar MC, Whitehurst P, Faivre FC (2019) The evolving role of radiotherapy in non-small cell lung cancer. Br J Radiol 92(1104):20190524. 10.1259/bjr.20190524 31535580 10.1259/bjr.20190524 PMC6913359

[CR7] Busch CJ, Kroger MS, Jensen J, Kriegs M, Gatzemeier F, Petersen C, Munscher A, Rothkamm K, Rieckmann T (2017) G2-checkpoint targeting and radiosensitization of HPV/p16-positive HNSCC cells through the inhibition of Chk1 and Wee1. Radiother Oncol 122(2):260–266. 10.1016/j.radonc.2016.11.017 27939202 10.1016/j.radonc.2016.11.017

[CR8] Carlos-Reyes A, Muniz-Lino MA, Romero-Garcia S, Lopez-Camarillo C, Hernandez-de LCO (2021) Biological adaptations of tumor cells to radiation therapy. Front Oncol 11:718636. 10.3389/fonc.2021.718636 34900673 10.3389/fonc.2021.718636 PMC8652287

[CR9] Chou CH, Huang MJ, Chen CH, Shyu MK, Huang J, Hung JS, Huang CS, Huang MC (2015) Up-regulation of C1GALT1 promotes breast cancer cell growth through MUC1-C signaling pathway. Oncotarget 6(8):6123–6135. 10.18632/oncotarget.3045 25762620 10.18632/oncotarget.3045 PMC4467426

[CR10] Chou CH, Huang MJ, Liao YY, Chen CH, Huang MC (2017) C1GALT1 Seems to promote in vitro disease progression in ovarian cancer. Int J Gynecol Cancer 27(5):863–871. 10.1097/IGC.0000000000000965 28498248 10.1097/IGC.0000000000000965

[CR11] Chugh S, Barkeer S, Rachagani S, Nimmakayala RK, Perumal N, Pothuraju R, Atri P, Mahapatra S, Thapa I, Talmon GA, Smith LM, Yu X, Neelamegham S, Fu J, Xia L, Ponnusamy MP, Batra SK (2018) Disruption of C1galt1 gene promotes development and metastasis of pancreatic adenocarcinomas in mice. Gastroenterology 155(5):1608–1624. 10.1053/j.gastro.2018.08.007 30086262 10.1053/j.gastro.2018.08.007 PMC6219903

[CR12] Denton EJ, Hart D, Wainer Z, Wright G, Russell PA, Conron M (2016) Changing trends in diagnosis, staging, treatment and survival in lung cancer: comparison of three consecutive cohorts in an Australian lung cancer centre. Intern Med J 46(8):946–954. 10.1111/imj.13132 27177600 10.1111/imj.13132

[CR13] Dong X, Luo Z, Wang Y, Meng L, Duan Q, Qiu L, Peng F, Shen L (2018) Altered *O*-glycosylation is associated with inherent radioresistance and malignancy of human laryngeal carcinoma. Exp Cell Res 362(2):302–310. 10.1016/j.yexcr.2017.11.031 29179977 10.1016/j.yexcr.2017.11.031

[CR14] Dong X, Chen C, Deng X, Liu Y, Duan Q, Peng Z, Luo Z, Shen L (2021a) A novel mechanism for C1GALT1 in the regulation of gastric cancer progression. Cell Biosci 11(1):166. 10.1186/s13578-021-00678-2 34452648 10.1186/s13578-021-00678-2 PMC8393437

[CR15] Dong X, Liu Y, Deng X, Shao J, Tian S, Chen S, Huang R, Lin Z, Chen C, Shen L (2021b) C1GALT1, negatively regulated by miR-181d-5p, promotes tumor progression via upregulating RAC1 in lung adenocarcinoma. Front Cell Dev Biol 9:707970. 10.3389/fcell.2021.707970 34307388 10.3389/fcell.2021.707970 PMC8292976

[CR16] Fu J, Wei B, Wen T, Johansson ME, Liu X, Bradford E, Thomsson KA, McGee S, Mansour L, Tong M, McDaniel JM, Sferra TJ, Turner JR, Chen H, Hansson GC, Braun J, Xia L (2011) Loss of intestinal core 1-derived *O*-glycans causes spontaneous colitis in mice. J Clin Invest 121(4):1657–1666. 10.1172/JCI45538 21383503 10.1172/JCI45538 PMC3069788

[CR17] Fu C, Zhao H, Wang Y, Cai H, Xiao Y, Zeng Y, Chen H (2016) Tumor-associated antigens: Tn antigen, sTn antigen, and T antigen. HLA 88(6):275–286. 10.1111/tan.12900 27679419 10.1111/tan.12900

[CR18] Gomez-Casal R, Bhattacharya C, Ganesh N, Bailey L, Basse P, Gibson M, Epperly M, Levina V (2013) Non-small cell lung cancer cells survived ionizing radiation treatment display cancer stem cell and epithelial-mesenchymal transition phenotypes. Mol Cancer 12(1):94. 10.1186/1476-4598-12-94 23947765 10.1186/1476-4598-12-94 PMC3751356

[CR19] Gu J, Lu C, Guo J, Chen L, Chu Y, Ji Y, Ge D (2013) Prognostic significance of the IASLC/ATS/ERS classification in Chinese patients—a single institution retrospective study of 292 lung adenocarcinoma. J Surg Oncol 107(5):474–480. 10.1002/jso.23259 22952152 10.1002/jso.23259

[CR20] Hanson RL, Hollingsworth MA (2016) Functional consequences of differential *O*-glycosylation of MUC1, MUC4, and MUC16 (downstream effects on signaling). Biomolecules 6(3):34. 10.3390/biom6030034 27483328 10.3390/biom6030034 PMC5039420

[CR21] Hay ED (2005) The mesenchymal cell, its role in the embryo, and the remarkable signaling mechanisms that create it. Dev Dyn 233(3):706–720. 10.1002/dvdy.20345 15937929 10.1002/dvdy.20345

[CR22] Hernandez-Vega AM, Del MA, Zamora-Sanchez CJ, Pina-Medina AG, Gonzalez-Arenas A, Camacho-Arroyo I (2020) Estradiol induces epithelial to mesenchymal transition of human glioblastoma cells. Cells 9(9):1930. 10.3390/cells9091930 32825553 10.3390/cells9091930 PMC7564468

[CR23] Huang Y, Hong W, Wei X (2022) The molecular mechanisms and therapeutic strategies of EMT in tumor progression and metastasis. J Hematol Oncol 15(1):129. 10.1186/s13045-022-01347-8 36076302 10.1186/s13045-022-01347-8 PMC9461252

[CR24] Hung JJ, Jeng WJ, Chou TY, Hsu WH, Wu KJ, Huang BS, Wu YC (2013) Prognostic value of the new International Association for the Study of Lung Cancer/American Thoracic Society/European Respiratory Society lung adenocarcinoma classification on death and recurrence in completely resected stage I lung adenocarcinoma. Ann Surg 258(6):1079–1086. 10.1097/SLA.0b013e31828920c0 23532112 10.1097/SLA.0b013e31828920c0

[CR25] Hung JJ, Yeh YC, Jeng WJ, Wu KJ, Huang BS, Wu YC, Chou TY, Hsu WH (2014a) Predictive value of the international association for the study of lung cancer/American Thoracic Society/European Respiratory Society classification of lung adenocarcinoma in tumor recurrence and patient survival. J Clin Oncol 32(22):2357–2364. 10.1200/JCO.2013.50.1049 24799473 10.1200/JCO.2013.50.1049

[CR26] Hung JS, Huang J, Lin YC, Huang MJ, Lee PH, Lai HS, Liang JT, Huang MC (2014b) C1GALT1 overexpression promotes the invasive behavior of colon cancer cells through modifying *O*-glycosylation of FGFR2. Oncotarget 5(8):2096–2106. 10.18632/oncotarget.1815 24758762 10.18632/oncotarget.1815 PMC4039148

[CR27] Jiang Y, Liu Z, Xu F, Dong X, Cheng Y, Hu Y, Gao T, Liu J, Yang L, Jia X, Qian H, Wen T, An G (2018a) Aberrant O-glycosylation contributes to tumorigenesis in human colorectal cancer. J Cell Mol Med 22(10):4875–4885. 10.1111/jcmm.13752 29999571 10.1111/jcmm.13752 PMC6156240

[CR28] Jiang YH, You KY, Bi ZF, Li LT, Mo HQ, Liu YM (2018b) The relationship between the radioresistance of pancreatic cancer cell SW1990 and the induction of the epithelial-mesenchymal transition: an in vitro study. Zhonghua Yi Xue Za Zhi 98(12):939–943. 10.3760/cma.j.issn.0376-2491.2018.12.013 29665670 10.3760/cma.j.issn.0376-2491.2018.12.013

[CR29] Josson S, Sharp S, Sung SY, Johnstone PA, Aneja R, Wang R, Gururajan M, Turner T, Chung LW, Yates C (2010) Tumor-stromal interactions influence radiation sensitivity in epithelial- versus mesenchymal-like prostate cancer cells. J Oncol 2010:232831. 10.1155/2010/232831 20798867 10.1155/2010/232831 PMC2926670

[CR30] Ju T, Cummings RD (2002) A unique molecular chaperone Cosmc required for activity of the mammalian core 1β3-galactosyltransferase. Proc Natl Acad Sci USA 99(26):16613–16618. 10.1073/pnas.262438199 12464682 10.1073/pnas.262438199 PMC139192

[CR31] Ju T, Brewer K, D’Souza A, Cummings RD, Canfield WM (2002a) Cloning and expression of human core 1β1,3-galactosyltransferase. J Biol Chem 277(1):178–186. 10.1074/jbc.M109060200 11677243 10.1074/jbc.M109060200

[CR32] Ju T, Cummings RD, Canfield WM (2002b) Purification, characterization, and subunit structure of rat core 1β1,3-galactosyltransferase. J Biol Chem 277(1):169–177. 10.1074/jbc.M109056200 11673471 10.1074/jbc.M109056200

[CR33] Ju T, Otto VI, Cummings RD (2011) The Tn antigen-structural simplicity and biological complexity. Angew Chem Int Ed Engl 50(8):1770–1791. 10.1002/anie.201002313 21259410 10.1002/anie.201002313 PMC7159538

[CR34] Kameda K, Eguchi T, Lu S, Qu Y, Tan KS, Kadota K, Adusumilli PS, Travis WD (2018) Implications of the eighth edition of the TNM proposal: invasive versus total tumor size for the T descriptor in pathologic stage I-IIA lung adenocarcinoma. J Thorac Oncol 13(12):1919-1929. 10.1016/j.jtho.2018.08.2022 10.1016/j.jtho.2018.08.2022 PMC6309787 30195703

[CR35] Kong FM, Zhao J, Wang J, Faivre-Finn C (2014) Radiation dose effect in locally advanced non-small cell lung cancer. J Thorac Dis 6(4):336–347. 10.3978/j.issn.2072-1439.2014.01.23 24688778 10.3978/j.issn.2072-1439.2014.01.23 PMC3968556

[CR36] Kuo TC, Wu MH, Yang SH, Chen ST, Hsu TW, Jhuang JY, Liao YY, Tien YW, Huang MC (2021) C1GALT1 high expression is associated with poor survival of patients with pancreatic ductal adenocarcinoma and promotes cell invasiveness through integrin alphav. Oncogene 40(7):1242–1254. 10.1038/s41388-020-01594-4 33420364 10.1038/s41388-020-01594-4 PMC7892338

[CR37] Lin MC, Chien PH, Wu HY, Chen ST, Juan HF, Lou PJ, Huang MC (2018) C1GALT1 predicts poor prognosis and is a potential therapeutic target in head and neck cancer. Oncogene 37(43):5780–5793. 10.1038/s41388-018-0375-0 29930379 10.1038/s41388-018-0375-0 PMC6202324

[CR38] Lin NY, Chen ST, Chang HL, Lu MY, Yang YL, Chou SW, Lin DT, Lin KH, Jou ST, Hsu WM, Huang MC, Chang HH (2022) C1GALT1 expression predicts a favorable prognosis and suppresses malignant phenotypes via TrkA signaling in neuroblastoma. Oncogenesis 11(1):8. 10.1038/s41389-022-00383-w 35169131 10.1038/s41389-022-00383-w PMC8847342

[CR39] Liu CH, Hu RH, Huang MJ, Lai IR, Chen CH, Lai HS, Wu YM, Huang MC (2014) C1GALT1 promotes invasive phenotypes of hepatocellular carcinoma cells by modulating integrin beta1 glycosylation and activity. PLoS ONE 9(8):e94995. 10.1371/journal.pone.0094995 25089569 10.1371/journal.pone.0094995 PMC4121071

[CR40] Liu F, Fu J, Bergstrom K, Shan X, McDaniel JM, McGee S, Bai X, Chen W, Xia L (2020) Core 1-derived mucin-type *O*-glycosylation protects against spontaneous gastritis and gastric cancer. J Exp Med 217(1):e20182325. 10.1084/jem.20182325 31645367 10.1084/jem.20182325 PMC7037257

[CR41] Lortet-Tieulent J, Soerjomataram I, Ferlay J, Rutherford M, Weiderpass E, Bray F (2014) International trends in lung cancer incidence by histological subtype: adenocarcinoma stabilizing in men but still increasing in women. Lung Cancer 84(1):13–22. 10.1016/j.lungcan.2014.01.009 24524818 10.1016/j.lungcan.2014.01.009

[CR42] Meza R, Meernik C, Jeon J, Cote ML (2015) Lung cancer incidence trends by gender, race and histology in the United States, 1973–2010. PLoS ONE 10(3):e0121323. 10.1371/journal.pone.0121323 25822850 10.1371/journal.pone.0121323 PMC4379166

[CR43] Montero-Calle A, Lopez-Janeiro A, Mendes ML, Perez-Hernandez D, Echevarria I, Ruz-Caracuel I, Heredia-Soto V, Mendiola M, Hardisson D, Argueso P, Pelaez-Garcia A, Guzman-Aranguez A, Barderas R (2023) In-depth quantitative proteomics analysis revealed C1GALT1 depletion in ECC-1 cells mimics an aggressive endometrial cancer phenotype observed in cancer patients with low C1GALT1 expression. Cell Oncol (dordr) 46(3):697–715. 10.1007/s13402-023-00778-w 36745330 10.1007/s13402-023-00778-w PMC10205863

[CR44] Nantajit D, Lin D, Li JJ (2015) The network of epithelial-mesenchymal transition: potential new targets for tumor resistance. J Cancer Res Clin Oncol 141(10):1697–1713. 10.1007/s00432-014-1840-y 25270087 10.1007/s00432-014-1840-y PMC4382462

[CR45] Nicholson AG, Tsao MS, Beasley MB, Borczuk AC, Brambilla E, Cooper WA, Dacic S, Jain D, Kerr KM, Lantuejoul S, Noguchi M, Papotti M, Rekhtman N, Scagliotti G, van Schil P, Sholl L, Yatabe Y, Yoshida A, Travis WD (2022) The 2021 WHO classification of lung tumors: impact of advances since 2015. J Thorac Oncol 17(3):362–387. 10.1016/j.jtho.2021.11.003 34808341 10.1016/j.jtho.2021.11.003

[CR46] Palma DA, Olson R, Harrow S, Gaede S, Louie AV, Haasbeek C, Mulroy L, Lock M, Rodrigues GB, Yaremko BP, Schellenberg D, Ahmad B, Griffioen G, Senthi S, Swaminath A, Kopek N, Liu M, Moore K, Currie S, Bauman GS, Warner A, Senan S (2019) Stereotactic ablative radiotherapy versus standard of care palliative treatment in patients with oligometastatic cancers (SABR-COMET): a randomised, phase 2, open-label trial. Lancet 393(10185):2051–2058. 10.1016/S0140-6736(18)32487-5 30982687 10.1016/S0140-6736(18)32487-5

[CR47] Pastushenko I, Blanpain C (2019) EMT transition states during tumor progression and metastasis. Trends Cell Biol 29(3):212–226. 10.1016/j.tcb.2018.12.001 30594349 10.1016/j.tcb.2018.12.001

[CR48] Qiao L, Chen Y, Liang N, Xie J, Deng G, Chen F, Wang X, Liu F, Li Y, Zhang J (2022) Targeting epithelial-to-mesenchymal transition in radioresistance: crosslinked mechanisms and strategies. Front Oncol 12:775238. 10.3389/fonc.2022.775238 35251963 10.3389/fonc.2022.775238 PMC8888452

[CR49] Qin Q, Cheng H, Lu J, Zhan L, Zheng J, Cai J, Yang X, Xu L, Zhu H, Zhang C, Liu J, Ma J, Zhang X, Dai S, Sun X (2014) Small-molecule survivin inhibitor YM155 enhances radiosensitization in esophageal squamous cell carcinoma by the abrogation of G2 checkpoint and suppression of homologous recombination repair. J Hematol Oncol 7:62. 10.1186/s13045-014-0062-8 25139395 10.1186/s13045-014-0062-8 PMC4237864

[CR50] Ritchie ME, Phipson B, Wu D, Hu Y, Law CW, Shi W, Smyth GK (2015) Limma powers differential expression analyses for RNA-sequencing and microarray studies. Nucleic Acids Res 43(7):e47. 10.1093/nar/gkv007 25605792 10.1093/nar/gkv007 PMC4402510

[CR51] Robinson MD, McCarthy DJ, Smyth GK (2010) edgeR: a bioconductor package for differential expression analysis of digital gene expression data. Bioinformatics 26(1):139–140. 10.1093/bioinformatics/btp616 19910308 10.1093/bioinformatics/btp616 PMC2796818

[CR52] Shi JF, Wang L, Wu N, Li JL, Hui ZG, Liu SM, Yang BY, Gao SG, Ren JS, Huang HY, Zhu J, Liu CC, Fan JH, Zhao SJ, Xing PY, Zhang Y, Li N, Lei WD, Wang DB, Huang YC, Liao XZ, Xing XJ, Du LB, Yang L, Liu YQ, Zhang YZ, Zhang K, Qiao YL, He J, Dai M (2019) Clinical characteristics and medical service utilization of lung cancer in China, 2005–2014: overall design and results from a multicenter retrospective epidemiologic survey. Lung Cancer 128:91–100. 10.1016/j.lungcan.2018.11.031 30642458 10.1016/j.lungcan.2018.11.031

[CR53] Stevens R, Macbeth F, Toy E, Coles B, Lester JF (2015) Palliative radiotherapy regimens for patients with thoracic symptoms from non-small cell lung cancer. Cochrane Database Syst Rev 1(1):CD002143. 10.1002/14651858.CD002143.pub4 25586198 10.1002/14651858.CD002143.pub4 PMC7017846

[CR54] Sun X, Zhan M, Sun X, Liu W, Meng X (2021) C1GALT1 in health and disease. Oncol Lett 22(2):589. 10.3892/ol.2021.12850 34149900 10.3892/ol.2021.12850 PMC8200938

[CR55] Sung H, Ferlay J, Siegel RL, Laversanne M, Soerjomataram I, Jemal A, Bray F (2021) Global cancer statistics 2020: GLOBOCAN estimates of incidence and mortality worldwide for 36 cancers in 185 countries. CA Cancer J Clin 71(3):209–249. 10.3322/caac.21660 33538338 10.3322/caac.21660

[CR56] Tan S, Yi P, Wang H, Xia L, Han Y, Wang H, Zeng B, Tang L, Pan Q, Tian Y, Rao S, Oyang L, Liang J, Lin J, Su M, Shi Y, Liao Q, Zhou Y (2020) RAC1 Involves in the radioresistance by mediating epithelial-mesenchymal transition in lung cancer. Front Oncol 10:649. 10.3389/fonc.2020.00649 32411607 10.3389/fonc.2020.00649 PMC7198748

[CR57] Tarp MA, Clausen H (2008) Mucin-type *O*-glycosylation and its potential use in drug and vaccine development. Biochim Biophys Acta 1780(3):546–563. 10.1016/j.bbagen.2007.09.010 17988798 10.1016/j.bbagen.2007.09.010

[CR58] Theys J, Jutten B, Habets R, Paesmans K, Groot AJ, Lambin P, Wouters BG, Lammering G, Vooijs M (2011) E-cadherin loss associated with EMT promotes radioresistance in human tumor cells. Radiother Oncol 99(3):392–397. 10.1016/j.radonc.2011.05.044 21680037 10.1016/j.radonc.2011.05.044 PMC4948667

[CR59] Tiwari N, Gheldof A, Tatari M, Christofori G (2012) EMT as the ultimate survival mechanism of cancer cells. Semin Cancer Biol 22(3):194–207. 10.1016/j.semcancer.2012.02.013 22406545 10.1016/j.semcancer.2012.02.013

[CR60] Tran DT, Ten HK (2013) Mucin-type *O*-glycosylation during development. J Biol Chem 288(10):6921–6929. 10.1074/jbc.R112.418558 23329828 10.1074/jbc.R112.418558 PMC3591602

[CR61] Travis WD, Brambilla E, Noguchi M, Nicholson AG, Geisinger KR, Yatabe Y, Beer DG, Powell CA, Riely GJ, Van Schil PE, Garg K, Austin JH, Asamura H, Rusch VW, Hirsch FR, Scagliotti G, Mitsudomi T, Huber RM, Ishikawa Y, Jett J, Sanchez-Cespedes M, Sculier JP, Takahashi T, Tsuboi M, Vansteenkiste J, Wistuba I, Yang PC, Aberle D, Brambilla C, Flieder D, Franklin W, Gazdar A, Gould M, Hasleton P, Henderson D, Johnson B, Johnson D, Kerr K, Kuriyama K, Lee JS, Miller VA, Petersen I, Roggli V, Rosell R, Saijo N, Thunnissen E, Tsao M, Yankelewitz D (2011) International association for the study of lung cancer/american thoracic society/european respiratory society international multidisciplinary classification of lung adenocarcinoma. J Thorac Oncol 6(2):244–285. 10.1097/JTO.0b013e318206a221 21252716 10.1097/JTO.0b013e318206a221 PMC4513953

[CR62] Travis WD, Asamura H, Bankier AA, Beasley MB, Detterbeck F, Flieder DB, Goo JM, MacMahon H, Naidich D, Nicholson AG, Powell CA, Prokop M, Rami-Porta R, Rusch V, van Schil P, Yatabe Y (2016) The IASLC lung cancer staging project: proposals for coding T categories for subsolid nodules and assessment of tumor size in part-solid tumors in the forthcoming eighth edition of the TNM classification of lung cancer. J Thorac Oncol 11(8):1204–1223. 10.1016/j.jtho.2016.03.025 10.1016/j.jtho.2016.03.025 27107787

[CR63] Tsutani Y, Miyata Y, Mimae T, Kushitani K, Takeshima Y, Yoshimura M, Okada M (2013) The prognostic role of pathologic invasive component size, excluding lepidic growth, in stage I lung adenocarcinoma. J Thorac Cardiovasc Surg 146(3):580–585. 10.1016/j.jtcvs.2013.04.032 23778085 10.1016/j.jtcvs.2013.04.032

[CR64] Tzeng SF, Tsai CH, Chao TK, Chou YC, Yang YC, Tsai MH, Cha TL, Hsiao PW (2018) *O*-Glycosylation-mediated signaling circuit drives metastatic castration-resistant prostate cancer. FASEB J. 10.1096/fj.201800687 29906246 10.1096/fj.201800687

[CR65] Vansteenkiste J, De Ruysscher D, Eberhardt WE, Lim E, Senan S, Felip E, Peters S (2013) Early and locally advanced non-small-cell lung cancer (NSCLC): ESMO clinical practice guidelines for diagnosis, treatment and follow-up. Ann Oncol 24(Suppl 6):vi89–vi98. 10.1093/annonc/mdt241 23860613 10.1093/annonc/mdt241

[CR66] Wan Y, Adair K, Herrmann A, Shan X, Xia L, Duckworth CA, Yu LG (2023) C1GalT1 expression reciprocally controls tumour cell-cell and tumour-macrophage interactions mediated by galectin-3 and MGL with double impact on cancer development and progression. Cell Death Dis 14(8):547. 10.1038/s41419-023-06082-7 37612278 10.1038/s41419-023-06082-7 PMC10447578

[CR67] Wang Y, Liao X, Ye Q, Huang L (2018) Clinic implication of MUC1 *O*-glycosylation and C1GALT1 in esophagus squamous cell carcinoma. Sci China Life Sci 61(11):1389–1395. 10.1007/s11427-017-9345-7 30076562 10.1007/s11427-017-9345-7

[CR68] Warth A, Muley T, Meister M, Stenzinger A, Thomas M, Schirmacher P, Schnabel PA, Budczies J, Hoffmann H, Weichert W (2012) The novel histologic International Association for the Study of Lung Cancer/American Thoracic Society/European Respiratory Society classification system of lung adenocarcinoma is a stage-independent predictor of survival. J Clin Oncol 30(13):1438–1446. 10.1200/JCO.2011.37.2185 22393100 10.1200/JCO.2011.37.2185

[CR69] Wu YM, Liu CH, Huang MJ, Lai HS, Lee PH, Hu RH, Huang MC (2013) C1GALT1 enhances proliferation of hepatocellular carcinoma cells via modulating MET glycosylation and dimerization. Cancer Res 73(17):5580–5590. 10.1158/0008-5472.CAN-13-0869 23832667 10.1158/0008-5472.CAN-13-0869

[CR70] Xia T, Xiang T, Xie H (2022) Update on the role of C1GALT1 in cancer. Oncol Lett 23(3):97. 10.3892/ol.2022.13217 35154428 10.3892/ol.2022.13217 PMC8822393

[CR71] Yang H, Wu L, Ke S, Wang W, Yang L, Gao X, Fang H, Yu H, Zhong Y, Xie C, Zhou F, Zhou Y (2016) Downregulation of ubiquitin-conjugating enzyme UBE2D3 promotes telomere maintenance and radioresistance of Eca-109 human esophageal carcinoma cells. J Cancer 7(9):1152–1162. 10.7150/jca.14745 27326259 10.7150/jca.14745 PMC4911883

[CR72] Yoshizawa A, Motoi N, Riely GJ, Sima CS, Gerald WL, Kris MG, Park BJ, Rusch VW, Travis WD (2011) Impact of proposed IASLC/ATS/ERS classification of lung adenocarcinoma: prognostic subgroups and implications for further revision of staging based on analysis of 514 stage I cases. Mod Pathol 24(5):653–664. 10.1038/modpathol.2010.232 21252858 10.1038/modpathol.2010.232

[CR73] Yoshizawa A, Sumiyoshi S, Sonobe M, Kobayashi M, Fujimoto M, Kawakami F, Tsuruyama T, Travis WD, Date H, Haga H (2013) Validation of the IASLC/ATS/ERS lung adenocarcinoma classification for prognosis and association with EGFR and KRAS gene mutations: analysis of 440 Japanese patients. J Thorac Oncol 8(1):52–61. 10.1097/JTO.0b013e3182769aa8 23242438 10.1097/JTO.0b013e3182769aa8

[CR74] Zhang Y, Weinberg RA (2018) Epithelial-to-mesenchymal transition in cancer: complexity and opportunities. Front Med 12(4):361–373. 10.1007/s11684-018-0656-6 30043221 10.1007/s11684-018-0656-6 PMC6186394

[CR75] Zhang C, Deng X, Qiu L, Peng F, Geng S, Shen L, Luo Z (2018) Knockdown of C1GalT1 inhibits radioresistance of human esophageal cancer cells through modifying beta1-integrin glycosylation. J Cancer 9(15):2666–2677. 10.7150/jca.25252 30087707 10.7150/jca.25252 PMC6072818

[CR76] Zhou S, Zhang M, Zhou C, Wang W, Yang H, Ye W (2020) The role of epithelial-mesenchymal transition in regulating radioresistance. Crit Rev Oncol Hematol 150:102961. 10.1016/j.critrevonc.2020.102961 32361589 10.1016/j.critrevonc.2020.102961

